# Metalloporphyrin/Phthalocyanine Catalysts for Electrocatalytic Nitrogen Fixation

**DOI:** 10.1002/advs.202509323

**Published:** 2025-09-11

**Authors:** Hai Sun, Shun Lu, Yuanyuan Qi, Jiahui Wu, Terence Liu, Jun‐Sheng Qin, Heng Rao

**Affiliations:** ^1^ State Key Laboratory of Inorganic Synthesis and Preparative Chemistry College of Chemistry International Center of Future Science Jilin University Changchun Jilin 130012 China; ^2^ Chongqing Institute of Green and Intelligent Technology Chinese Academy of Sciences Chongqing 400714 China; ^3^ Department of Mechanical and Construction Engineering Northumbria University Newcastle upon Tyne NE1 8ST UK

**Keywords:** C─N coupling reaction, electrocatalytic nitrogen fixation, porphyrin/phthalocyanine complexes, reaction mechanism

## Abstract

Metalloporphyrin/phthalocyanine complexes serve as excellent electrocatalytic models due to their well‐defined MN_4_, structural tunability, and chemical stability. They have been applied in many catalytic fields and have shown excellent catalytic activity, such as oxygen reduction reaction (ORR) and carbon dioxide reduction reaction (CO_2_RR), etc. The application of these complexes in electrocatalytic nitrogen fixation, such as nitrogen reduction reactions (NRR), nitrate reduction reactions (NO_3_RR), nitrite reduction reactions (NO_2_RR), and nitrate/nitrite/nitrogen‐carbon dioxide co‐reduction (N_2_/NO_x_‐CO_2_RR) for urea production, has been emphasized in recent years. The main obstacles for these complexes to function effectively as electrocatalysts involve the necessity for a well‐thought‐out design of porphyrin/phthalocyanine structures and a more comprehensive understanding of their structure‐activity relationships. This review summarizes the modifying porphyrin/phthalocyanine complex methods, constructing derivative strategies, and exploring the relevant electrocatalytic reaction mechanisms and their applications for electrocatalytic nitrogen fixation. Optimization strategies based on reaction mechanisms and catalytic performance are proposed. Finally, this comprehensive overview aims to provide professional insights that will guide future experimental and theoretical advancements in porphyrin/phthalocyanine complexes and highlight their challenges and prospects for electrocatalytic systems.

## Graphical abstract

This review focuses on the application of metal porphyrin/phthalocyanine‐based catalysts in electrocatalytic nitrogen fixation. The detailed examination encompasses the structure and modification strategies of metal porphyrin/phthalocyanine‐based catalysts, their application in electrocatalytic nitrogen fixation, and the associated reaction mechanisms. Optimization strategies are proposed based on the structure‐activity relationship.

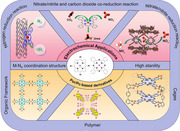

## 1 Introduction

**Figure 1:**
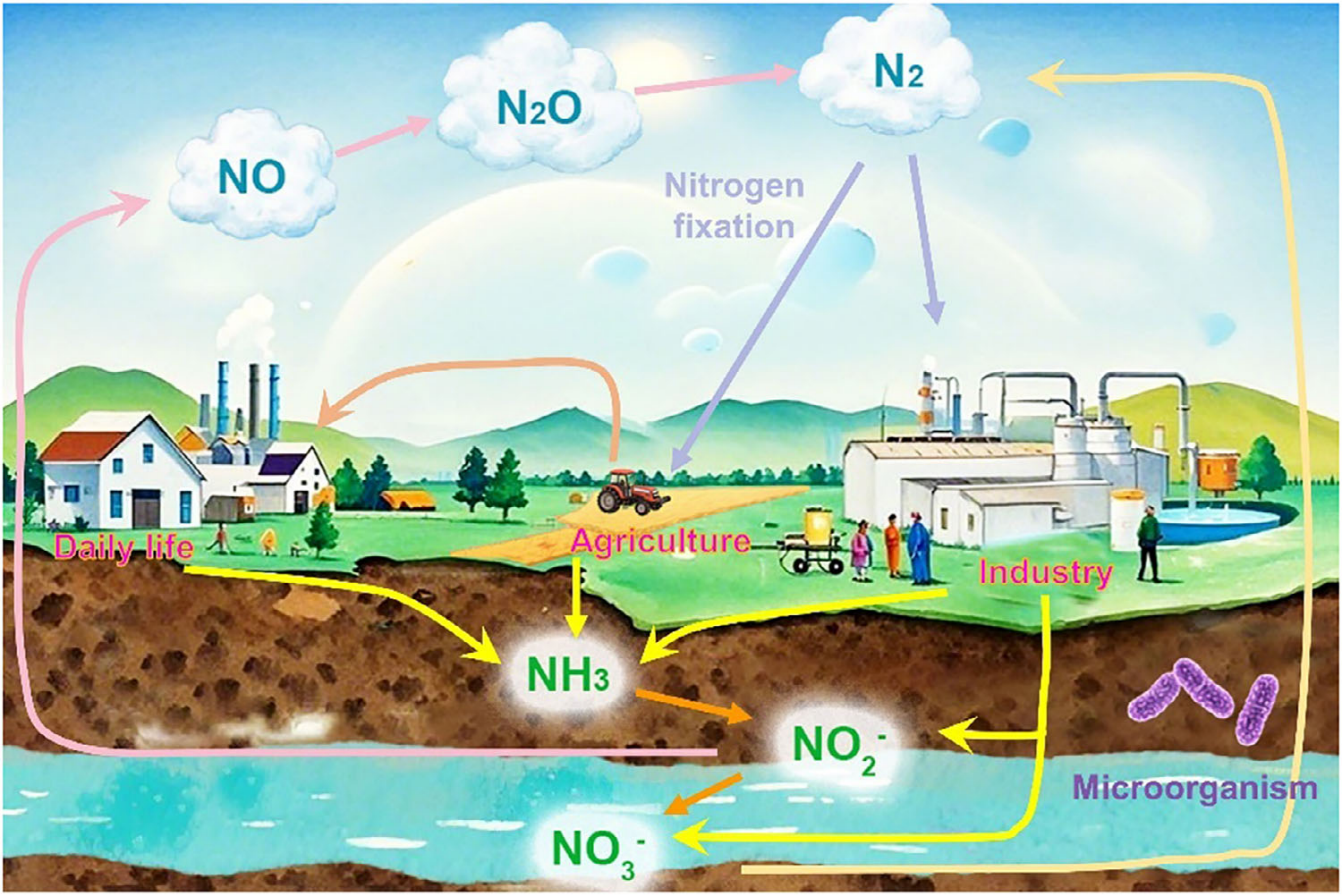
Nitrogen cycle network in nature.

Nitrogen (N) is a basic element for living organisms, which determines its irreplaceable role.^[^ ^1^ ^]^ N exists on Earth in various forms, such as nitrogen gas (N_2_), nitric oxide (NO), nitrogen dioxide (NO_2_), nitrates (NO_3_^−^), nitrites (NO_2_^−^), ammonia (NH_3_), and urea (NH_2_CONH_2_). N_2_ is the main form of nitrogen, accounting for 78% of the air. The concentration of nitrogen within the Earth's crust is notably low, constituting approximately 0.0046% of the total crustal composition.^[^ ^2^ ^]^ The stability of nitrogen is attributed to the presence of the N≡N (941 kJ·mol^−1^).^[^ ^3^ ^]^ This makes it difficult for most plants and animals on Earth to directly convert and utilize N_2_. Only a small number of bacteria, microorganisms, and enzymes can directly utilize N_2_. Under natural conditions, N_2_ can be converted into active nitrogen by lightning, which is beneficial to plants and animals. Most plants and animals can only transform or absorb more reactive nitrogen compounds such as NH_3_, NH_2_CONH_2_, and NO_3_^−^.^[^ ^4^ ^]^ Most of the nitrogen‐containing substances on Earth can naturally be converted into each other, but this process is intricate, involving nitrogen fixation, nitrification, denitrification, and ammonification, as illustrated in **Figure** **1**.^[^ ^5^ ^]^ The interconversion between nitrogen‐containing substances ensures the nitrogen cycle. The nitrogen cycle constitutes a crucial element of Earth's material cycles, significantly contributing to the maintenance of ecological equilibrium.^[^ ^6^ ^]^

With rapid global development and explosive population growth, humans have caused serious damage to the ecological environment.^[^ ^7^ ^]^ Rapid population growth requires additional food resources. Nitrogen compounds are important nutrients for crops and can effectively increase crop yield. However, the nitrogen compounds that exist in nature and can be directly used by plants do not meet the needs of agricultural production. Therefore, artificial nitrogen fixation is required to meet the needs of agricultural development. The Haber–Bosch process has been used to synthesize NH_3_ on a large scale since 1913.^[^ ^8^ ^]^ However, the process requires harsh reaction conditions and consumes a lot of energy.^[^ ^9^ ^]^ Currently, non‐renewable energy sources continue to serve as the primary raw materials for energy supply. The combustion of fossil fuels also releases toxic gases (NO, N_2_O, and SO_2_, etc.), causing substantial damage to the ecological environment and affecting human survival and development.^[^ ^10^ ^]^ While nitrogen fertilizers meet agricultural needs on a large scale, they have effectively doubled the nitrogen input into both terrestrial and marine ecosystems. These active nitrogen substances that are not effectively utilized spread rapidly with water flow, resulting in extensive growth of plankton and plants in the water. The massive reproduction of algae not only causes aquatic animals to lose their living space but also breeds a large number of bacteria. Moreover, active nitrogen substances in water can enter the human body through water intake and directly affect human health.^[^ ^11^ ^]^ For instance, excessive nitrite intake can convert hemoglobin in the human body into iron hemoglobin, which has no oxygen‐carrying capacity. Eventually leading to diseases such as respiratory failure. Additionally, NO_2_^−^ and NO_3_^−^ exhibit carcinogenic effects. Therefore, there is an urgent need to convert excess N‐containing substances to protect the environment and human health. Among the many nitrogen conversion technologies, electrocatalytic technology has shown great application potential due to its mild reaction conditions, controllable reactions, and high conversion rate.^[^ ^12^ ^]^ At present, electrocatalytic technology has been effectively implemented in the nitrogen reduction reaction (NRR)^[^ ^13^ ^]^ as well as in the nitrate and nitrite reduction reactions (NOxRR),^[^ ^14^ ^]^ and has also achieved satisfactory results. However, electrocatalytic technology is still restricted by many factors, such as temperature, electrolyte, and catalyst, among which the catalyst is a particularly important factor.^[^ ^15^ ^]^ Catalysts can affect both the catalytic efficiency and product distribution. Currently, catalysts still face problems, such as high price, poor stability, and low efficiency, which restrict their commercial applications. Given these challenges, efficient catalysts with high catalytic efficiency, good stability, and excellent product selectivity are urgently required.^[^ ^16^ ^]^

Catalysts are capable of significantly lowering the energy barrier of reactions, thereby facilitating their progression. Therefore, catalyst design is particularly important. Numerous catalysts composed of non‐precious metals exhibiting high catalytic activity have been developed. In recent years, metal nitrogen carbon (MNC) materials have become a focal point of research interest due to their unique electronic configurations, efficient atomic utilization, remarkable catalytic properties, and outstanding stability.^[^ ^17^ ^]^ Metalloporphyrin (MPor) and metal phthalocyanine (MPc) molecular catalysts are ideal materials for studying structure‐activity relationships and catalytic mechanisms because of their clear MN_4_ structure, unique electronic properties, and directional synthesis.^[^ ^18^ ^]^ However, the poor conductivity of MPor/Pc results in only moderate catalytic activity. In addition, Por/Pc molecules tend to aggregate during the catalytic process because of the interactions between molecules, which ultimately reduces their electron transfer ability and catalytic activity. To address these issues, researchers have employed various methods to avoid this situation. For example, introducing a carrier can not only improve the conductivity but also delay the agglomeration effect.^[^ ^19^ ^]^ Integrating Por/Pc molecules into an organic framework can prevent aggregation effectively.^[^ ^20^ ^]^ This has greatly facilitated the advancement and utilization of Por/Pc catalysts. The utilization of Por/Pc catalysts in electrocatalytic nitrogen fixation had garnered significant attention from researchers until 2020. Catalysts based on Por/Pc have demonstrated significant catalytic activity and exhibit substantial potential for further research.^[^ ^21^ ^]^ Consequently, we concentrate on summarizing the design of Por/Pc‐based catalysts, the reaction mechanism of electrocatalytic nitrogen fixation, the structure‐activity relationship, and the main challenges and solutions. We anticipate that this review will encourage the utilization of Por/Pc‐based catalysts in the field of electrocatalytic nitrogen fixation.

## 2 Modification and Construction of Porphyrin/Phthalocyanine Catalysts

Previous studies have shown that MPor and MPc are excellent catalysts, and their catalytic activities can be significantly improved by targeted modifications.^[^ ^22^ ^]^ Considering the utilization of Por/Pc‐based catalysts within the domain of electrocatalysis and the influence of modification techniques on their catalytic performance, we summarized the commonly used modification methods for Por/Pc‐based catalysts and discussed the effects of these modification methods on the electrocatalytic nitrogen fixation. Currently, several common methods are available for modifying Por/Pc‐based catalysts. The first is to modify Por/Pc at the molecular level, such as by modifying substituents.^[^ ^23^ ^]^ The second approach involves utilizing Por/Pc as a foundational element for the synthesis of organic frameworks,^[^ ^24^ ^]^ including covalent organic frameworks (COF),^[^ ^25^ ^]^ among others. Because Por/Pc‐based materials have the disadvantage of poor electrical conductivity, some researchers have generated metal–nitrogen–carbon materials from Por/Pc‐based materials via calcination.^[^ ^26^ ^]^ In the following section, we discuss various modification methods and their effects on catalytic performance.

### 2.1 Design of Porphyrin and Phthalocyanine Molecules

**Figure 2:**
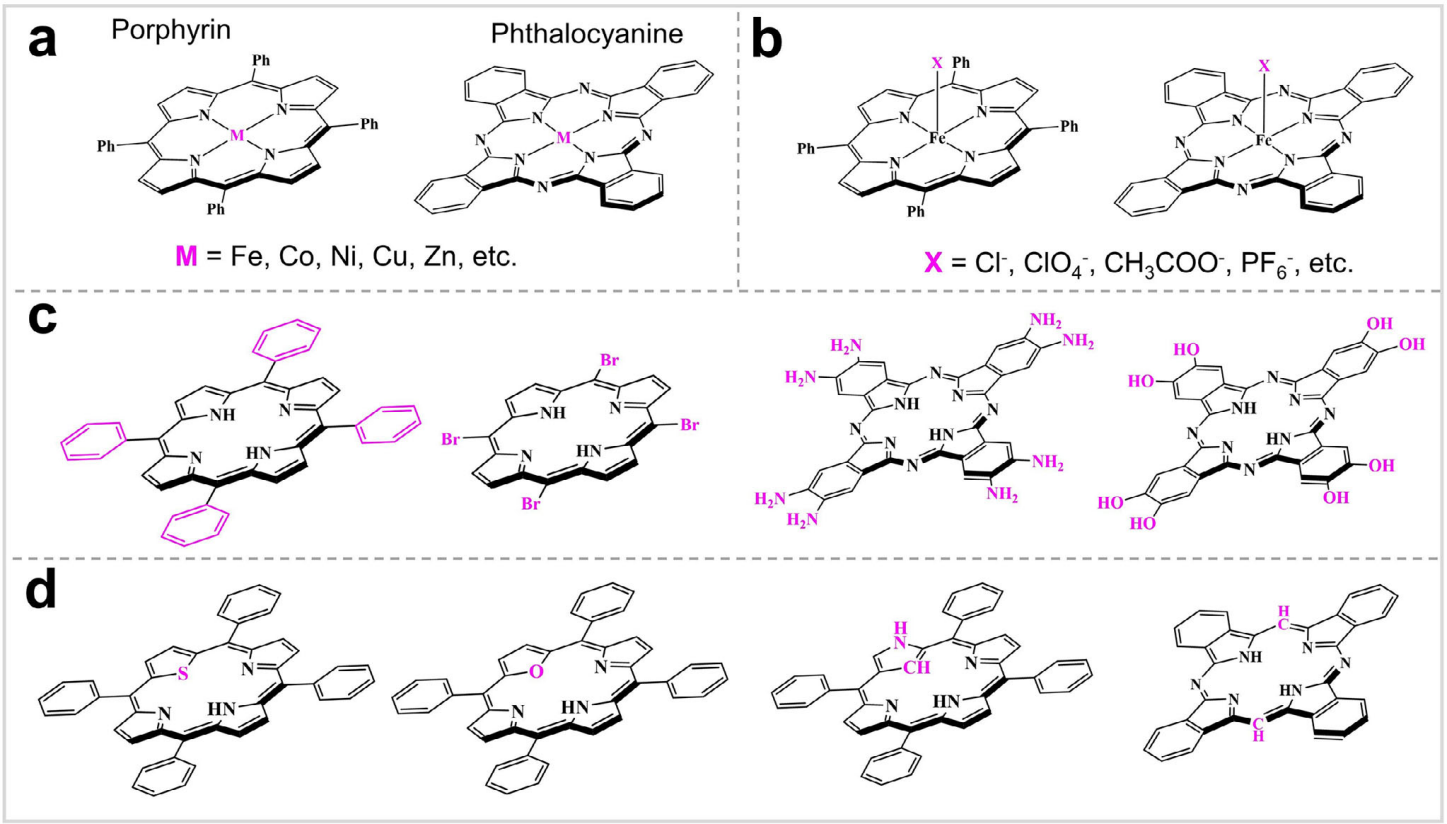
(a) Structures of MPor and MPc with different metals. (b) Structures of MPor and MPc with different coordinated anions. (c) Structures of Por and Pc with different substituents. (d) Structures of Por and Pc with different inner ring structures.

Por and Pc molecules are extensively utilized in electrocatalysis due to their well‐defined and tunable structures. By examining the correlation between structural characteristics and catalytic activity, it is possible to identify the catalytic site and further investigate the reaction mechanism.^[^ ^27^ ^]^ Currently, there are four main methods for modifying Por/Pc molecular catalysts (**Figure** **2**): 1) Substituting the central metal atoms, such as Fe, Cu, Ni, Zn, and other metals. 2) Changing the axially coordinated anions of the macrocycle. 3) Modifying functional groups, such as acid/base groups. 4) Changing the inner ring structure, such as MN_3_O_1_, MN_3_S_1_, MN_2_O_2_, and MN_2_S_2_.

The catalytic site of MPor/MPc is usually considered to be the metal at the center of the macrocycle because of the different adsorption and conversion abilities of metals on reaction substrates. Sakata et al. synthesized tetraphenyl metalloporphyrins (MTPP) (M = Co, Ni, Fe, Zn, Cu, Mg, and Mn) and loaded them onto gas diffusion electrodes to study their ability to reduce CO_2_.^[^ ^28^ ^]^ The study found that the reduction ability of CoTPP and FeTPP for CO_2_ was significantly higher than that of CuTPP and ZnTPP. Liang et al. synthesized MPc with different metals, with Co, Ni, Zn, Cu, and Mn, and evaluated their catalytic activity for ORR.^[^ ^29^ ^]^ The experiments and theoretical calculation results show that ZnPc and CoPc have better catalytic activities than the other MPcs. These studies show that changing the metal centers of MPc and MPor is an effective way to regulate their catalytic activities. The axially coordinated anions of MPor/MPc are also key factors affecting the catalytic activity. Masaoka et al. first synthesized tetraphenylporphyrin iron (III) chloride (FeTPP‐Cl) and then synthesized FeTPP‐ClO_4_ by replacing Cl^−^ with ClO_4_^−^.^[^ ^30^ ^]^ They found that the solubility of FeTPP‐ClO_4_ in acetonitrile (MeCN) was greatly improved compared with FeTPP‐Cl. Meanwhile, the turnover frequency (TOF) of FeTPP‐ClO_4_ for CO_2_RR in acetonitrile solution was 7.3×10^6^ S^−1^, which is much larger than that of FeTPP‐Cl in DMF solution (1.1×10^2^ S^−1^). In order to elucidate this phenomenon, the researchers conducted a comprehensive examination of the reaction mechanism. In MeCN, Fe^II^/Fe^I^ of FeTPP are catalytic centers for CO_2_, whereas in DMF, the catalytic center of FeTPP for CO_2_ shifts to Fe^I^/Fe^0^. These studies have shown that the axially coordinated anions of MPor can significantly alter the solubility and reaction path of MPor, thus affecting its catalytic activity. Modifying the substituents on MPor and MPc significantly altered their catalytic activities. Different substituents play different roles in the catalytic process. For example, charged substituents not only influence the charge distributions of MPor/MPc but also have a stabilizing effect on intermediates. Savéant modified substituents with different electron‐attracting capacities onto FePor and tested their catalytic activity for CO_2_RR under homogeneous conditions.^[^ ^31^ ^]^ As the substituent's electron‐attracting capability increases, the overpotential of the corresponding FePor for CO_2_RR also decreases. The substituent exhibits a stronger electron‐attracting capability, which reduces the charge of Fe in FePor, thereby facilitating the adsorption of CO_2_. Nyokong et al. synthesized CoPc(X)_4_ with different substituents (X = COOH, NO_2_, C(CH_3_)_3_, NH_2_, and SO_3_H).^[^ ^32^ ^]^ In an electrolyte at pH 8.3, the decreases in the catalytic current associated with cysteine oxidation are observed in the following order: COOH > C(CH_3_)_3_ > SO_3_H > NH_2_ > NO_2_. In addition to the above method to design porphyrin and phthalocyanine, Zhuang et al. successfully synthesized a porphyrin with an N_3_C_1_ structure by inverting the pyrrole in the porphyrin ring.^[^ ^33^ ^]^ Choi et al. synthesized a porphyrin with an N_3_O_1_ structure by replacing the N atom in the porphyrin ring with an O atom.^[^ ^34^ ^]^ The above studies have shown that modifying Por/Pc at the molecular level can significantly alter its catalytic activity in electrocatalytic reactions. Some researchers have modified MPor/MPc by the above method and tested them for electrocatalytic nitrogen fixation. We discuss specific research in detail in the corresponding chapters of this review.

### 2.2 Porphyrin/Phthalocyanine‐Based Organic Frameworks

**Figure 3:**
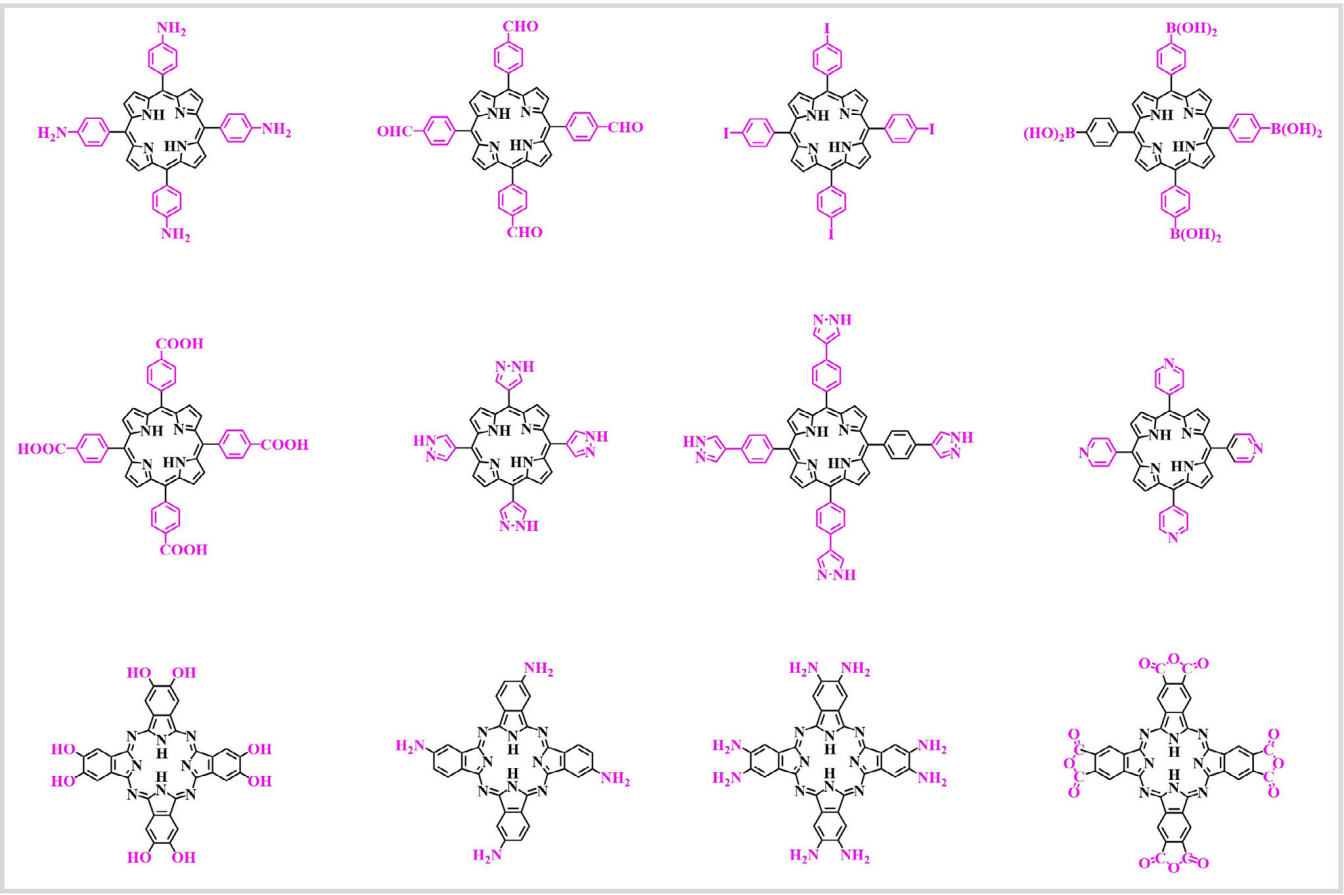
Structures of Por and Pc for synthesizing POFs.

In addition to serving as molecular catalysts, MPor and MPc can also be used as building blocks to construct organic frameworks with periodic structures.^[^ ^35^ ^]^ Metal–organic frameworks (MOFs) and covalent organic frameworks (COFs) containing Por/Pc are collectively referred to as Por/Pc organic frameworks (POFs).^[^ ^36^ ^]^ However, not all Pors and Pcs can be used to synthesize POFs. Only MPc and MPor, which possess specific functional groups such as carboxyl, amino, and hydroxyl groups, are suitable for the synthesis of POFs, as illustrated in **Figure** **3**.

In the realm of electrocatalysis, POFs are highly regarded by researchers due to their periodic network architecture, expansive specific surface area, modifiable pore dimensions, and the distinctive M‐N_4_ coordination framework they exhibit. Compared with MPc and MPor molecules, POFs can inhibit aggregation during the catalytic process, thereby exposing more active sites and ultimately achieving the purpose of increasing catalytic activity. Subsequently, we will classify and introduce POFs according to their different structures.

**Figure 4:**
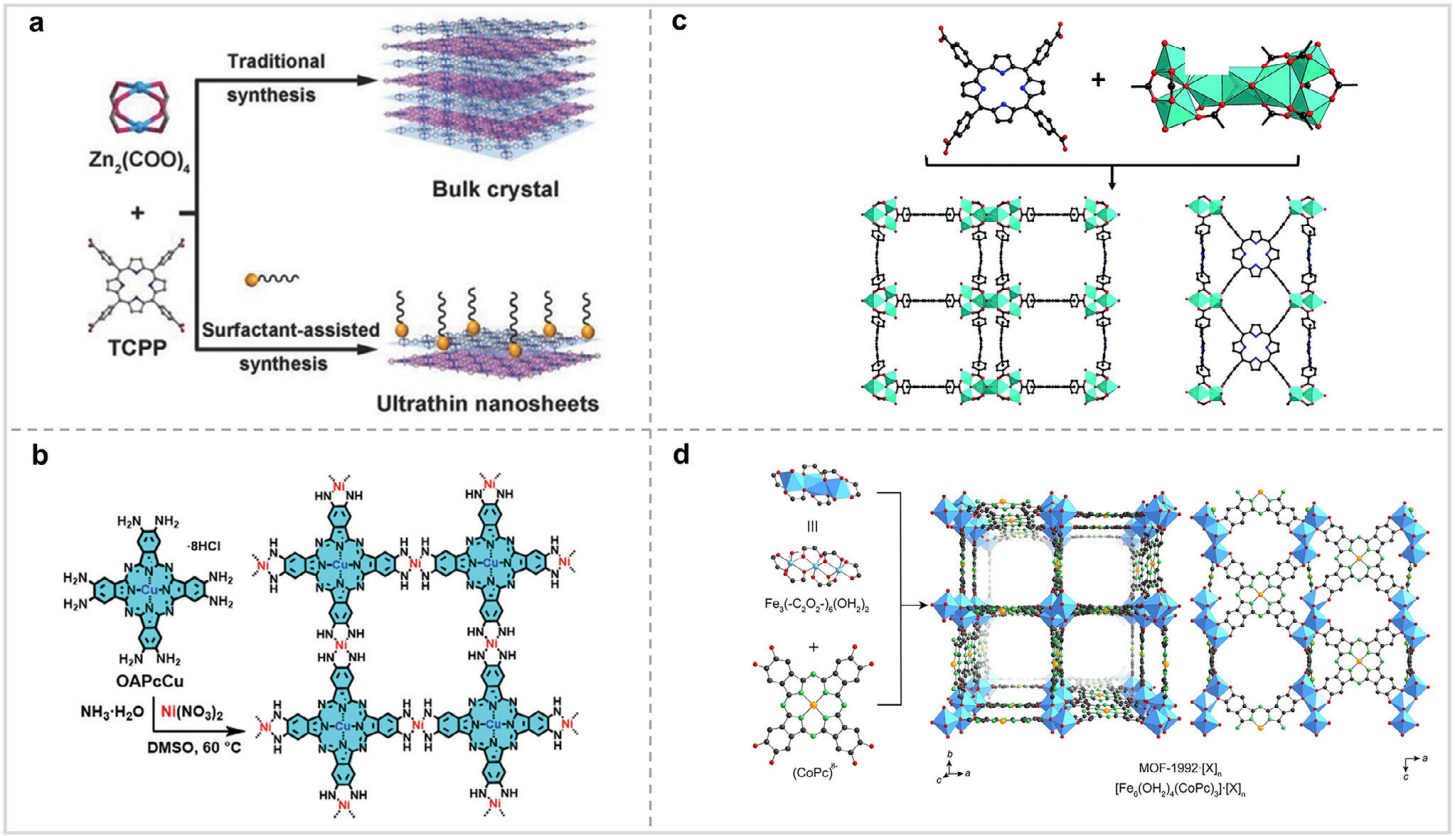
(a) Structure of Por‐MOF with 2D structure. Reproduced with permission.^[^ ^38^ ^]^ Copyright 2015, Wiley‐VCH GmbH. (b) Structure of Pc‐MOF with 2D structure. Reproduced with permission.^[^ ^39^ ^]^ Copyright 2020, Wiley‐VCH GmbH. (c) Structure of Por‐MOF with 3D structure. Reproduced with permission.^[^ ^41^ ^]^ Copyright 2015, Royal Society of Chemistry. (d) Structure of Pc‐MOF with 3D structure. Reproduced with permission.^[^ ^42^ ^]^ Copyright 2019, American Chemical Society.

MOFs are new crystalline porous materials connected by organic ligands and metal ions or metal clusters.^[^ ^37^ ^]^ MOFs exhibit significant porosity and well‐defined active sites, rendering them highly potential for electrocatalytic applications. In the process of electrocatalytic nitrogen fixation, substrates containing nitrogen can more effectively interact with the active sites, thereby enhancing catalytic activity. Therefore, the construction of MOFs significantly influences electrocatalytic nitrogen fixation. MOFs can be classified as two‐dimensional or three‐dimensional MOFs. Zhang et al. synthesized bulk M‐TCPP MOFs using tetrakis(4‐carboxyphenyl)porphyrin (TCPP) and metal salts as building blocks.^[^ ^38^ ^]^ Upon the incorporation of polyvinylpyrrolidone into the reaction mixture, ultrathin 2D M‐TCPP MOFs (where M represents Zn, Cu, Cd, or Co) were successfully synthesized, as depicted in **Figure** **4**a. The findings indicated that the Zn‐TCPP MOF lateral dimension was 1.2 ± 0.4 µm, the thickness of the single‐layer nanosheets was 7.6 ± 2.6 nm, the theoretical interlayer distance was 0.93 nm, and the number of layers was approximately 8 ± 3. The Brunauer–Emmett–Teller (BET) surface area for the bulk Zn‐TCPP MOF is quantified at 197 m^2^ g^−1^, and the BET surface area of the Zn‐TCPP nanosheet is 391 m^2^ g^−1^. In comparison to the bulk Zn‐TCPP MOF, the Zn‐TCPP MOF nanosheet demonstrates a greater specific surface area. Under the same circumstances, MOFs with large specific surface areas are more likely to expose active sites, thereby promoting catalytic reactions. Feng et al. used 2,3,9,10,16,17,23,24‐octa amino‐phthalocyanine copper [II] and nickel nitrate hexahydrate as precursors to synthesize bulk Ni_2_[CuPc(NH)_8_] 2D MOF (Figure 4b).^[^ ^39^ ^]^ Subsequently, bulk MOF and sodium chloride (NaCl) were added to the grinding bowl and exfoliated by ball milling to obtain ultrathin MOF nanosheets. The ultrathin MOF nanosheets exhibited an average lateral dimension of approximately 160 nm, with an average thickness of approximately 7 nm, corresponding to roughly 10 layers. Simultaneously, the BET surface area of the MOF nanosheets was 690 m^2^ g^−1^. Ni_2_[CuPc(NH)_8_] 2D MOF nanosheets have dual metal sites, which are more favorable for electrocatalytic nitrogen fixation. This is due to the fact that N has multiple valence states, the bimetallic sites are able to form a tandem effect to promote the reaction. Therefore, the design of 2D Por/Pc‐based MOFs with ultrathin structures and multiple active sites is beneficial for improving the electrocatalytic activity. Three‐dimensional metal–organic frameworks (3D MOFs) are more prevalently applied in electrocatalysis compared to their two‐dimensional counterparts (2D MOFs).^[^ ^40^ ^]^ 3D MOFs have richer pore structures, and their specific surface areas are usually larger than those of 2D MOFs, which is more conducive to the contact between the catalytic center and the reaction substrate. In recent years, most 3D Por/Pc‐MOFs have used metal ions or metal clusters with high‐valence states as metal junctions (Zr^4+^, Hf^4+^, Al^3+^, Fe^3+^, Cr^3+^). Zhou's group synthesized PCN‐22 with a 3D structure using titanium‐oxo clusters and TCPP as precursors via a solvothermal method (Figure 4c).^[^ ^41^ ^]^ The BET surface area of PCN‐22 was 1284 m^2^ g^−1^, and its pore diameter was 1.5 nm. Yaghi's group synthesized an anionic 3D MOF (MOF‐1992) with CoPc as precursors (Figure 4d).^[^ ^42^ ^]^ The BET surface area of MOF‐1992·[Fe]_3_ was 1471 m^2^ g^−1^. By comparing the BET surface areas of 2D Por/Pc‐MOF and 3D Por/Pc‐MOF, it was found that the BET surface area of 3D Por/Pc‐MOF was generally larger than that of 2D Por/Pc‐MOF. A large specific surface area is more favorable for electrocatalytic reactions because the active sites are easily exposed. Consequently, the development of 3D Por/Pc‐MOFs with extensive specific surface areas and robust structures represents a promising area of research.

**Figure 5:**
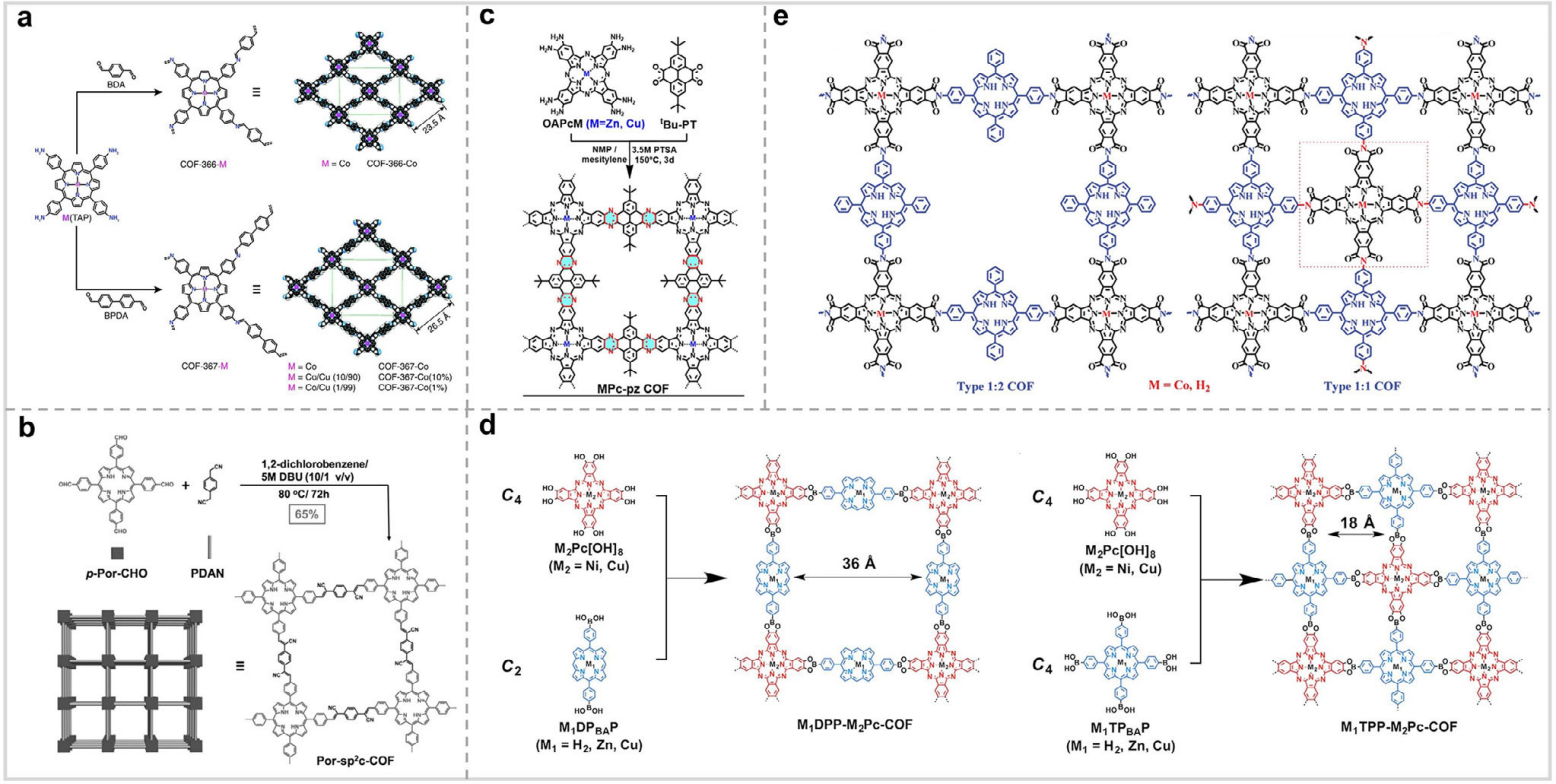
(a) Synthetic routes and schematics of COF‐366‐M and COF‐367‐M.^[^ ^46^ ^]^ Copyright 2015, American Association for the Advancement of Science. (b) Synthesis routes of Por‐sp^2^c‐COF. Reproduced with permission.^[^ ^47^ ^]^ Copyright 2019, Wiley‐VCH GmbH. (c) Design and synthesis of MPc‐pz COF. Reproduced with permission.^[^ ^48^ ^]^ Copyright 2019, American Chemical Society. (d) M_1_TPP‐M_2_Pc‐COFs and M_1_DPP‐M_2_Pc‐COFs structural diagrams. Reproduced with permission.^[^ ^49^ ^]^ Copyright 2016, Springer Nature. (e) Schematic diagram of COFs with different Por and Pc ratios. Reproduced with permission.^[^ ^50^ ^]^ Copyright 2022, Wiley‐VCH GmbH.

COFs are a crystalline material formed by covalent bonds between organic ligands.^[^ ^43^ ^]^ The formation of covalent bonds contributes to the enhanced physicochemical stability of COFs. The advantages of molecular catalysts and porous materials are combined in Por/Pc‐COFs, which makes them very promising for applications in electrocatalytic reactions. For example, the active sites of COFs using Por/Pc as building blocks are very uniformly distributed.^[^ ^44^ ^]^ Por/Pc‐COFs can also be expanded and optimized without changing the framework structure.^[^ ^45^ ^]^ Diercks's group used CoPor‐NH_2_ to connect different organic ligands through an imine bond to synthesize COF‐366‐M and COF‐367‐M, respectively (**Figure** **5**a).^[^ ^46^ ^]^ The imine bond was confirmed by infrared spectroscopy. The BET surface areas of COF‐366‐Co (1360 m^2^ g^−1^) and COF‐367‐Co (1470 m^2^ g^−1^) were more than 1000 m^2^ g^−1^. The COF frame was destroyed when the temperature exceeded 300 °C. COF‐366‐M and COF‐367‐M not only had large BET surface areas but also exhibited good stability. Wang et al. used Por‐CHO and 1,4‐phenylenediacetonitrile to synthesize a novel Por‐sp^2^c‐COF connected by C═C bonds (Figure 5b).^[^ ^47^ ^]^ The COF linked by C═C is more stable than that linked by imine bonds. The Por‐sp^2^c‐COF also maintained good stability in 9 M HCl and 9 M NaOH solutions. The poor conductivity of Por/Pc‐based catalysts limits their development for electrocatalysis. Therefore, it is particularly important to improve the conductivity of Por/Pc‐based catalysts through structural design. Feng et al. used MPcs (M = Zn and Cu) as ligands to generate two new Pc‐ COFs (MPc‐Pz COF) connected by pyrazine and studied the relationship between the structure and conductivity (Figure 5c).^[^ ^48^ ^]^ The MPc‐Pz COFs were p‐type semiconductors, and the intrinsic conductivity reached ≈5 × 10^−7^ S cm^−1^. COF with high conductivity can significantly improve the reaction efficiency. Therefore, studying the relationship between the COF structure and conductivity can greatly promote the application of COF in electrochemistry. MPc and MPs can also be integrated simultaneously into the same COF. Jiang's group reported a highly ordered 2D COF (M_1_DPP‐M_2_Pc‐COFs, M_1_TPP‐M_2_Pc‐COFs) synthesized from Por and Pc, which merges covalent bonds and non‐covalent forces (Figure 5d).^[^ ^49^ ^]^ They were formed by covalently polymerizing Por and Pc into two‐dimensional sheets, followed by non‐covalent crystallization. The M_1_DPP‐M_2_Pc‐COFs composed of C_4_ and C_2_ had a pore size of 36 Å, and the M_1_TPP‐M_2_Pc‐COFs composed of C_4_ and C_4_ had a pore size of 18 Å. Peng et al. synthesized two COFs with different Por and Pc ratios through structural design (Figure 5e).^[^ ^50^ ^]^ The ratios of Por and Pc in the two COFs are 1:1 and 1:2, respectively. In electrocatalytic nitrogen fixation, neutral or alkaline electrolytes are usually used. This requires a catalyst with good catalytic activity and stability. Por/Pc‐based COFs, which are linked by covalent bonds, not only have good stability but also retain the advantages of molecular catalysts. Therefore, Por/Pc‐COFs are promising electrocatalysts for electrocatalytic nitrogen fixation.

### 2.3 Porphyrin/Phthalocyanine‐Based Polymers

**Figure 6:**
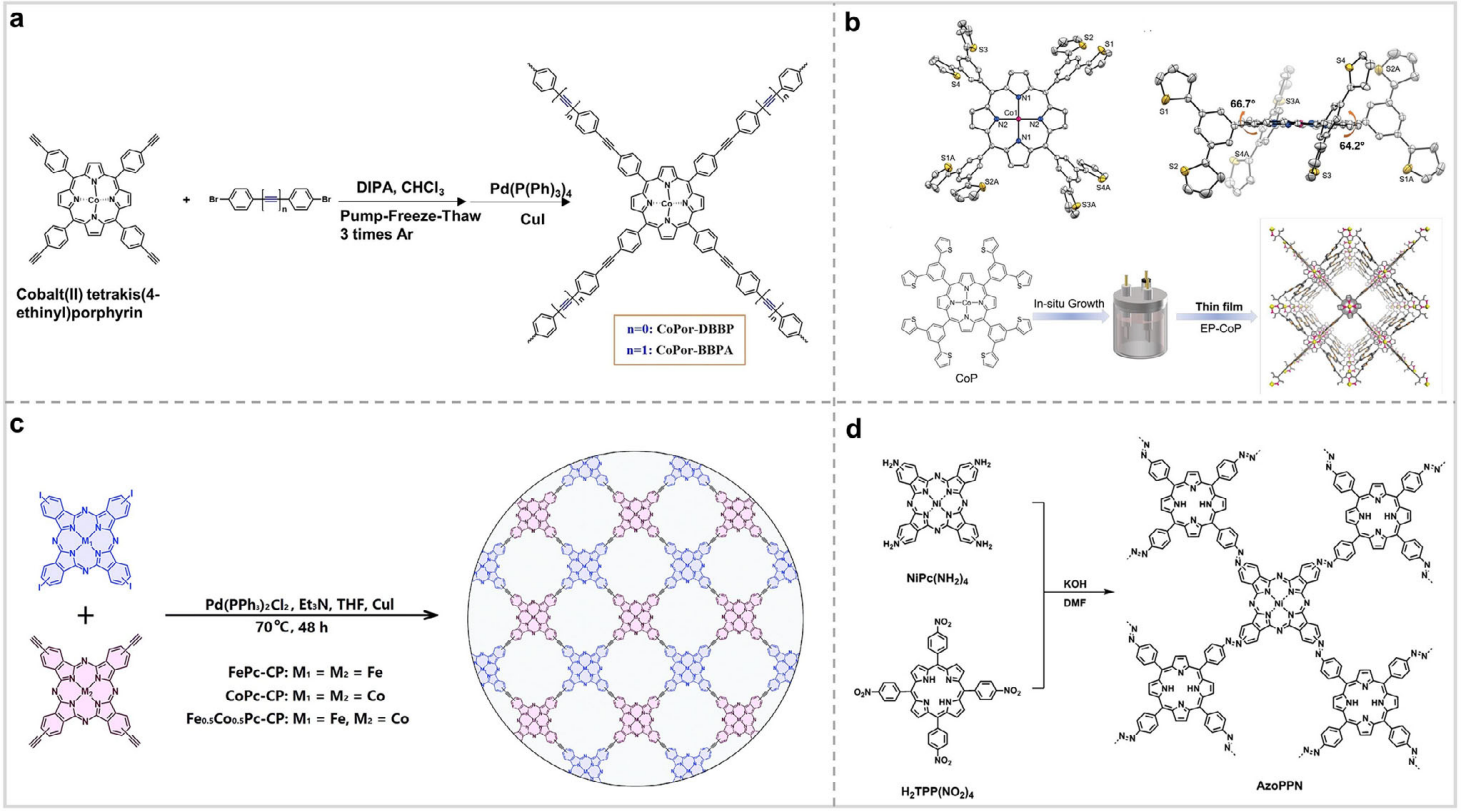
(a) Acetylene‐bridged CoPor‐polymer synthesis route. Reproduced with permission.^[^ ^52^ ^]^ Copyright 2022, Wiley‐VCH GmbH. (b) The synthetic route of EP‐CoP. Reproduced with permission.^[^ ^53^ ^]^ Copyright 2023, Wiley‐VCH GmbH. (c) Structural diagram of MPc‐MCPs. Reproduced with permission.^[^ ^54^ ^]^ Copyright 2018, Royal Society of Chemistry. (d) Synthesis route and structure of AzoPPN. Reproduced with permission.^[^ ^55^ ^]^ Copyright 2016, Wiley‐VCH GmbH.

Because Por/Pc has multiple functional groups, it can not only synthesize POFs with a crystal structure but also generate polymers with clear active sites through self‐polymerization or reactions with other ligands. The structural configuration of Por/Pc‐polymers closely resembles that of Por/Pc‐COFs, with the organic ligands being interconnected through covalent bonds.^[^ ^51^ ^]^ Therefore, Por/Pc‐polymers also have clear active sites and good stabilities. Song et al. synthesized Por‐polymers (CoPor‐DBBP and CoPor‐BBPA) linked by acetylene bridging units using the Sonogashira cross‐coupling reaction (**Figure** **6**a).^[^ ^52^ ^]^ The BET surface areas of CoPor‐DBBP and CoPor‐BBPA were 646 and 572 m^2^ g^−1^, respectively, and they all have microporous structures. Their work shows that the BET surface area of the Por‐polymers can be effectively adjusted by adjusting the ligand length. Por/Pc‐polymers can also be synthesized by electrocatalytic techniques. Zhu's group polymerized CoPor onto the surface of carbon nanotubes (CNTs) by electropolymerization, ultimately obtaining a 3D microporous nanofilm EP‐CoP (Figure 6b).^[^ ^53^ ^]^ Polymerizing Por‐COF onto the surface of CNTs can increase the bonding strength between COF and CNTs and shorten the charge transfer distance, which was also confirmed by their electrochemical test results. Yao's group synthesized a Pc‐polmer connected by acetylene bonds (Fe_0.5_Co_0.5_Pc‐CP) with dual metal sites (Figure 6c).^[^ ^54^ ^]^ Fe_0.5_Co_0.5_Pc‐CP is a noncrystalline material with an extended π‐conjugated network. Fe and Co were alternately distributed in the polymer network structure. The experimental results showed that the two metals were evenly distributed, and one metal was surrounded by four adjacent metals of the other metal. The two metals play a synergistic role in catalytic reactions. This polymer with dual metal sites, well‐defined structure, and good chemical stability has great potential for electrochemical applications. Different from the previous method, Han et al. synthesized a polymer (AzoPPN) by using H_2_TPP(NO_2_)_4_ and NiPc(NH_2_)_4_ as ligands (Figure 6d).^[^ ^55^ ^]^ Uniform staggered distribution of Por and Pc in AzoPPN. The BET surface area of AzoPPN was 400 m^2^ g^−1^, indicating that it has permanent micropores. The above studies show that the BET surface area, pore size, and active site distribution of Por/Pc‐polymers can be effectively adjusted by regulating the type and length of ligands, which provides good conditions for the application of Por/Pc‐polymers in electrocatalysis.

### 2.4 Porphyrin/Phthalocyanine‐Based Cages

**Figure 7:**
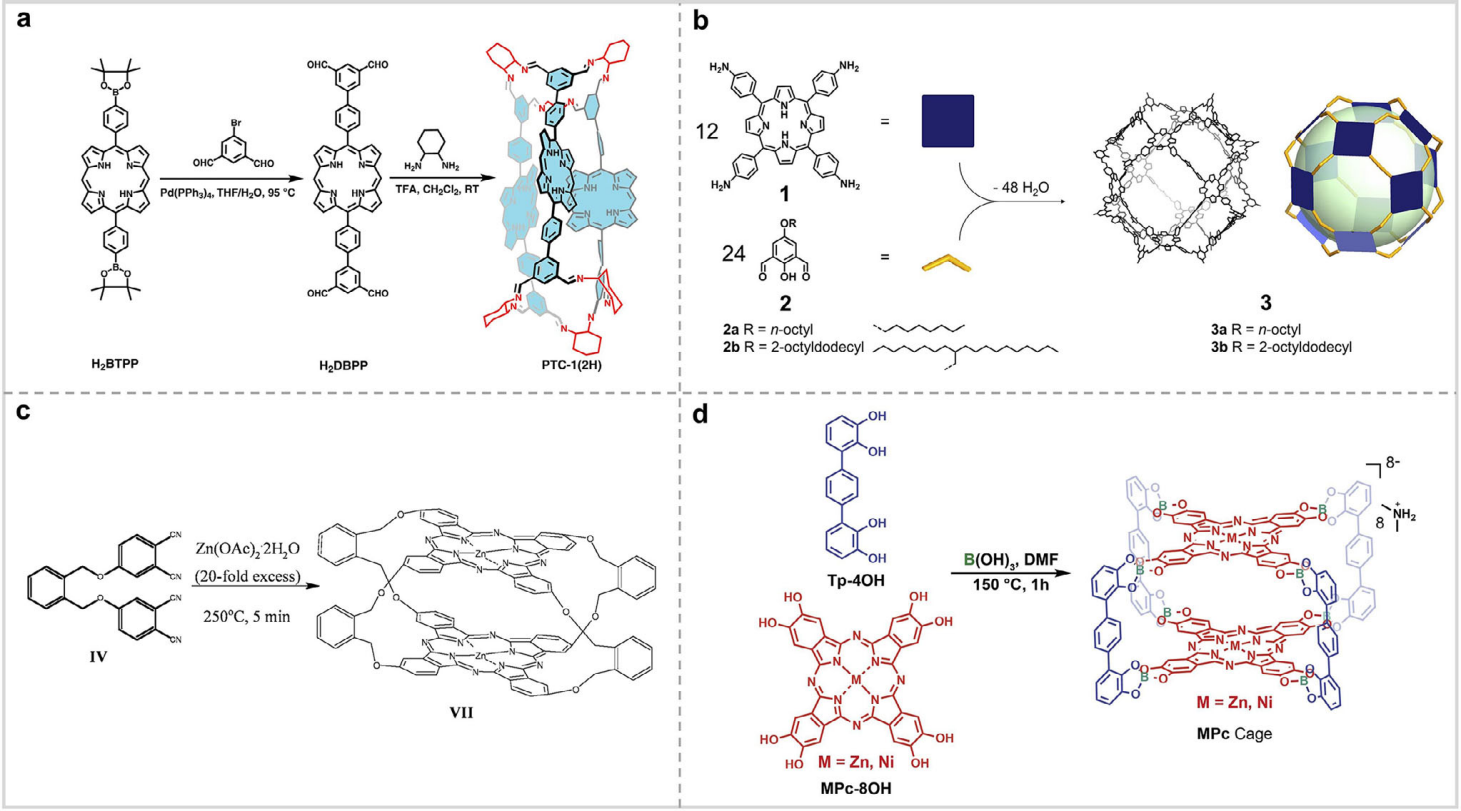
(a) PTC‐1(2H) synthetic route. Reproduced with permission.^[^ ^58^ ^]^ Copyright 2020, Springer Nature. (b) Design and synthesis of the Por Cages P_12_L_24_. Reproduced with permission.^[^ ^59^ ^]^ Copyright 2020. Elsevier. (c) Synthesis of binuclear Pc ball type. Reproduced with permission.^[^ ^61^ ^]^ Copyright 2003, World Scientific. (d) Schematic diagram of Pc cage synthesis. Reproduced with permission.^[^ ^62^ ^]^ Copyright 2023, Elsevier.

Organic cages, also known as covalent organic polyhedrons (COPs), have 3D structures and certain sized cavities.^[^ ^56^ ^]^ Por/Pc‐based metal–organic cages are excellent optoelectronic materials, which combine the excellent photophysical properties of Por/Pc with the host‐guest chemistry advantages of metal–organic cages.^[^ ^57^ ^]^ This gives it great application potential in the field of photoelectrocatalysis. Jiang et al. developed a Por tubular organic cage (PTC‐1(2H)) consisting of three porphyrin molecules and six cyclohexane diamine groups.(**Figure** **7**a).^[^ ^58^ ^]^ Each PTC‐1(2H) has a 3.3 nm tubular structure, and the BET surface area was 112 m^2^ g^−1^. Kim et al. have effectively synthesized and engineered a Por‐based organic cage, which possesses an outer diameter measuring 5.3 nm. (Figure 7b).^[^ ^59^ ^]^ This organic cage is composed of 36 building blocks connected by 48 covalent bonds. Long‐pillar connectors were installed precisely in the cage to demonstrate the benefits of a large cavity. This shows that this organic cage with a huge imaginary structure can encapsulate the required substances as required. This makes this Por‐based organic cage show great potential in many fields. Compared to Pors, Pcs have a planar structure and strong intermolecular π‐π interactions, which lead to their easy aggregation and reduced active site utilization during the reaction.^[^ ^60^ ^]^ Integrating phthalocyanine into a rigid organic cage can greatly improve the above problems. Nonetheless, the synthesis of Pc‐based organic cages encounters considerable obstacles due to the low solubility of Pc. Tomilova and colleagues synthesized a Pc‐based organic cage composed of two Pcs and four linkers (Figure 7c).^[^ ^61^ ^]^ The authors simply characterized it by ^1^H NMR, mass spectrometry, and UV‐visible absorption spectroscopy but did not conduct in‐depth research. Lu et al. reported a MPc cage linked by boronate bonds (Figure 7d).^[^ ^62^ ^]^ Single‐crystal X‐ray diffraction characterization revealed that the MPc cage contains two Pc molecules and four side arms. The distance between the two Pc molecules in the cage was 7.9 Å, and the distance between the Pc cages was 7.3 Å. The two Pc molecules covered each other almost perfectly, indicating that the Pc cage had good rigidity. Compared to Por/Pc molecules, the Por/Pc‐based cages not only preserve the advantages of molecular catalysts but also inhibit the aggregation effect of molecular catalysts, which is very beneficial for electrocatalytic reactions. Simultaneously, the cavities in the Por/Pc‐based organic cages can also anchor and encapsulate other substances, which greatly enhances their application effect and scope.

### 2.5 Porphyrin/Phthalocyanine‐Based Metal–Nitrogen–Carbon Materials

Metal–nitrogen–carbon materials (MNCs) have significant advantages in electrocatalysis (such as CO_2_ reduction, NO_3_^−^ reduction, and urea synthesis). Their core advantages come from the unique atomic‐level active site and tunable electronic/geometric properties. MNCs also have high stability and excellent charge transfer ability. MPor/Pc‐based catalysts with well‐defined active sites and MN_4_ structures are excellent precursors for the synthesis of MNCs. Although MNCs have shown good development potential, they also face many challenges. Firstly, pyrolysis will lead to poor uniformity of active sites of MNCs. This is because pyrolysis usually produces a mixture of MN_4_ sites, metal nanoparticles, and carbonaceous byproducts. Furthermore, the choice of metal, nitrogen, and carbon precursors affects the final structure, making reproducibility difficult at scale. Meantime, MNCs are affected by precursors, resulting in poor control over porosity and morphology. Scalable pyrolysis methods (e.g., tube furnaces) often yield materials with poorly defined pore networks, limiting mass transport in electrochemical applications. In addition, pyrolysis temperature is also an important factor affecting the catalytic effect of MNCs. At high pyrolysis temperatures, excessive graphitization can reduce defect density, while insufficient temperatures may leave unstable carbon structures prone to oxidation. Although the synthesis of MNCs still faces many challenges, they have shown good development potential in electrocatalysis. Therefore, it is necessary to develop MNCs with uniform active sites, good reproducibility, and controllable structure and morphology.

## 3 Nitrogen Reduction Reaction (NRR)

N_2_ is an important component of the atmosphere, accounting for about 78% of the atmosphere. N_2_ is also an important part of the nitrogen cycle.^[^ ^63^ ^]^ Denitrifying bacteria in nature can convert nitrogen oxides into N_2_, which can be converted into other nitrogen‐containing compounds. Currently, N_2_ can participate in the nitrogen cycle through natural transformation under natural conditions and artificial transformation. There are two forms of N_2_ conversion in nature. One method is to convert N_2_ into nitrogen compounds through the nitrogen fixation of bacteria or enzymes, which is the main form of N_2_ conversion in nature. The other is that N_2_ and O_2_ react to form nitrogen oxides during discharge.^[^ ^5c^ ^]^ Artificial nitrogen fixation is mainly achieved through the Haber‐Bosch process. However, the process requires a large amount of energy. At present, fuels are still the main energy supply material. High energy‐consuming industries not only emit harmful gases, such as SO_2_ and CO_2_, but also consume a lot of energy and cause an energy crisis.^[^ ^64^ ^]^ At present, it is urgent to seek technologies with mild conditions, high conversion efficiency, and no pollution to replace high‐energy consumption industries. Due to the advantages of electrocatalysis technology, researchers have applied electrocatalysis to NRR. However, due to the unclear reaction mechanism and catalyst limitations, electrocatalytic nitrogen reduction technology is still a long way from commercial application. Therefore, it is essential to study the reaction mechanism and design a catalyst that meets the required requirements.^[^ ^65^ ^]^

### 3.1 NRR Reaction Mechanism

**Figure 8:**
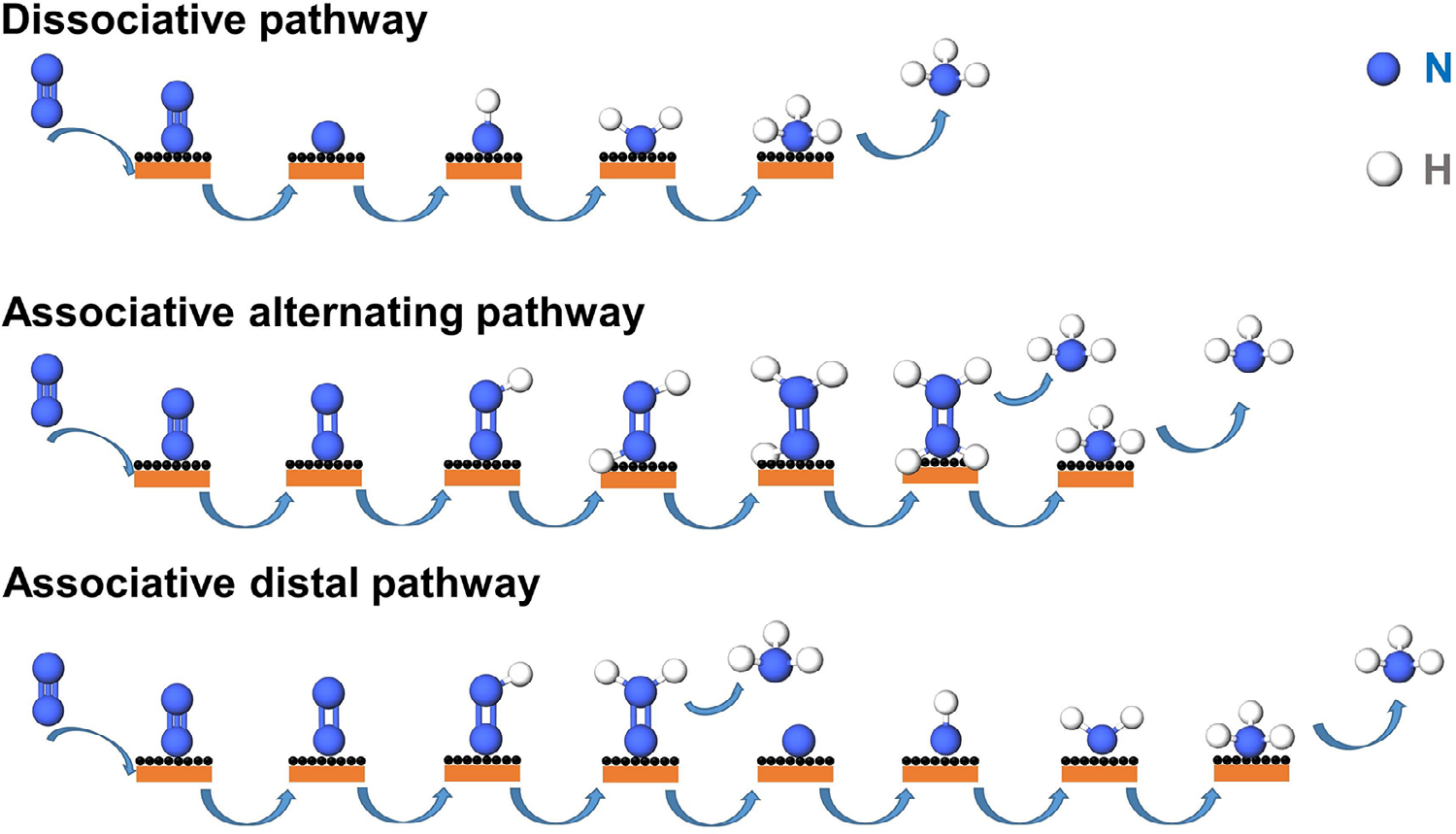
Mechanism of electrocatalytic nitrogen reduction reaction.

Many researchers have studied the NRR, and their results show that NH_3_ is the main product. Therefore, we mainly summarize the reduction of N_2_ to NH_3_ by Por/Pc‐based electrocatalysts in this section. In recent years, various characterization techniques have been developed rapidly, providing strong technical support for analyzing intrinsic changes in reactions. Since NRR involves multiple intermediates, NRR has multiple reaction pathways.^[^ ^66^ ^]^ At present, researchers have divided the electrocatalytic reduction of N_2_ to NH_3_ into three pathways based on the different modes of hydrogenation and nitrogen‐nitrogen bond breaking: dissociative, associative distal, and associative alternating.^[^ ^67^ ^]^ The NRR pathway is illustrated in **Figure** **8**. In the dissociation pathway, N≡N in *N_2_ is directly broken to generate two *N (* indicates the adsorption state). *N obtains three electrons and three protons to generate *NH_3_, and *NH_3_ detaches from the catalyst's surface to producing NH_3_. In the associative alternating pathway, the N≡N bond of N_2_ adsorbed on the catalyst will first be broken to form *N═N. The two N in *N═N alternately obtain electrons/protons to generate *H_3_NNH_3_, and finally, the NN bond breaks to generate two NH_3_. The associative distal pathway and the associative alternating pathway are slightly different. In the associative distal pathway, the terminal N in *N═N preferentially obtains three protons and three electrons to generate the *N═NH_3_ species. The N═N bond in *N═NH_3_ breaks to release one NH_3_ to form *N. *N continuously obtains three electrons and three protons to generate NH_3_.

### 3.2 Porphyrin Molecules for NRR

**Figure 9:**
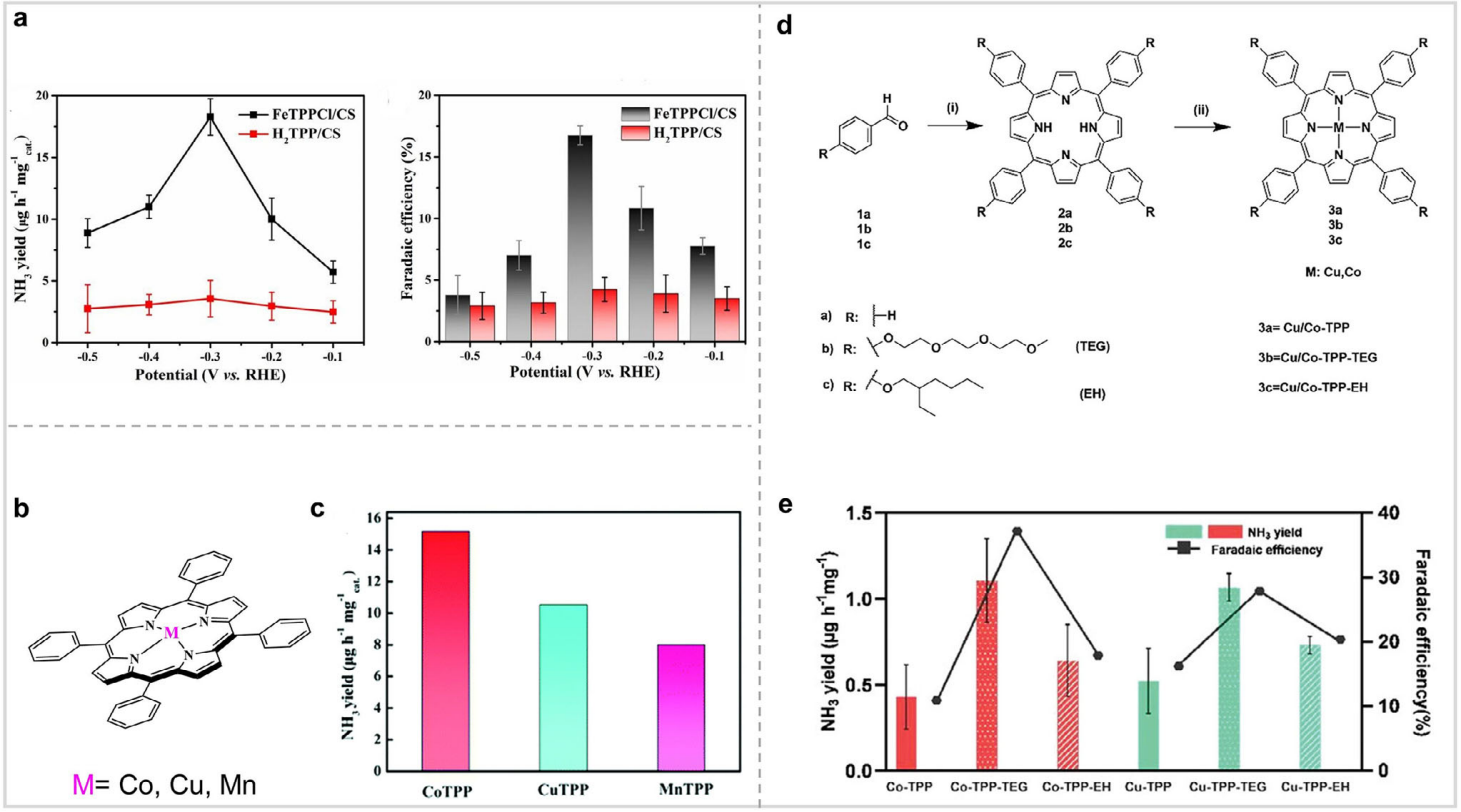
(a) Electrolysis performance of FeTPPCl/CS and H_2_TPP/CS for NRR. Reproduced with permission.^[^ ^69^ ^]^ Copyright 2021, Elsevier. (b) MTPP (M = Co, Cu, Mn) structural diagram. (c) Average NH_3_ yield of MTPP in 0.1M HCl electrolyte. Reproduced with permission.^[^ ^70^ ^]^ Copyright 2021, Royal Society of Chemistry. (d) Cu/Co‐TPP synthesis pathway. (e) NH_3_ yields and FEs of catalysts. Reproduced with permission.^[^ ^71^ ^]^ Copyright 2024, Wiley‐VCH GmbH.

MPor, as a structurally well‐defined and chemically stable organometallic complex, has shown great research potential in NRR. Tang et al. used MoPor as a model and calculated its catalytic activity for NRR based on first‐principles calculations.^[^ ^68^ ^]^ The calculation results showed that the charge distribution of MoPor changes, and more delocalized π electrons are activated after the Mo atom is anchored on the Por molecule. The associative distal pathway is identified as the optimal route for NRR using MoPor as a catalyst, and the overpotential is only 0.22 V. The authors assert that the excellent catalytic performance of MoPor is primarily owing to the highly localized and spin‐polarized Mo‐4d states. The aforementioned theoretical investigations have demonstrated that MPor serves as a highly effective catalyst for NRR, thereby establishing a theoretical foundation for the utilization of MPor in NRR applications. In nature, biological enzymes convert N_2_ to NH_3_. Studies have found that the Fe sites in biological enzymes can strongly interact with N_2_ to activate N_2_. Inspired by the bio‐enzymatic nitrogen fixation, Wu's group studied the electrocatalytic activity of tetraphenylporphyrin ferric chloride (FeTPPCl) with a clear FeN_4_ structure for NRR.^[^ ^69^ ^]^ FeTPPCl demonstrated superior catalytic performance, with an NH_3_ yield rate of 18.28 ± 1.6 µg h^−1^ mg^−1^ and a Faradaic efficiency (FE) of 16.76 ± 0.9% at −0.3 V versus RHE.(**Figure** **9**a). The authors have determined that the desorption of Cl^−^ from FeTPPCl is a critical step for facilitating the NRR. Theoretical studies have shown that [Fe(TPP)]^2−^ is the active species and that NRR undergoes an associative alternating pathway. Wu et al. demonstrated that MPors have good catalytic activity for NRR through experimental and theoretical calculations. The metal in MPor is also an important factor affecting its catalytic effect. Therefore, Zang et al. used cobalt tetraphenylporphyrin (CoTPP) as an electrocatalyst to study its catalytic performance for NRR (Figure 9b,c).^[^ ^70^ ^]^ Electrolysis results show the NH_3_ yield rate is 15.18±0.78 µg h^−1^ mg^−1^, the NH_3_ FE is 11.43 ± 0.74%. They replaced Co with Cu and Mn to synthesize CuTPP and MnTPP, respectively. The maximum NH_3_ yield of CuTPP was 10.53 ± 6.5 µg h^−1^ mg^−1^, and the maximum NH_3_ FE was 14.97%. The maximum NH_3_ yield of MnTPP is 7.98 ±0.8 µg h^−1^ mg^−1^, and the maximum NH_3_ FE is 8.21%. Compared with CuTPP and MnTPP, CoTPP exhibits better catalytic activity for NRR. This also shows that it is still an effective strategy to regulate the catalytic activity for NRR by changing the central metal of MPor. Although MPor has been shown to be an excellent catalyst for NRR, the hydrophobicity and low conductivity of MPor limit its development in the field of electrocatalysis. Modification of the substituents on Por can change charge distribution and hydrophilicity, thus affecting its catalytic effect. Therefore, modification of substituents on Por is considered an effective strategy for modulating its catalytic activity. Based on the above considerations, Abbotto et al. modified the hydrophilicity of Por by modifying the substituents and studied their catalytic activity for NRR (Figure 9d,e).^[^ ^71^ ^]^ The contact angles of Cu/Co‐TPP and Cu/Co‐TPP‐EH modified with hydrophobic substituents were both higher than 110°. The contact angles of Cu/Co‐TPP‐TEG modified with hydrophilic substituents were 29° and 39°, respectively. The above results show that it is effective to change the hydrophilicity of Por by modifying its substituents. The catalytic efficiency of MPor in promoting the NRR was assessed within an acidic electrolyte medium. The test results showed that Co/Cu‐TPP‐TEG showed the best catalytic performance, with NH_3_ yield and FE of 1.10 ± 0.07 µg h^−1^ mg^−1^_cat_ and 37%, respectively. This also shows that the modification of hydrophilic substituents is effective in improving the catalytic activity of MPor. The authors explain that the presence of hydrophilic groups can increase the interaction between the active center and the reaction substrate, ultimately achieving the purpose of improving catalytic performance. The studies referenced above indicate that the catalytic performance of MPor in the context of NRR can be significantly influenced by alterations to the metal center and the modification of functional groups. Researchers have systematically investigated the catalytic activity of MPor as a NRR catalyst until 2020. Therefore, there are fewer research projects, but the potential for MPor development has been confirmed for NRR.

### 3.3 Porphyrin‐Derived Materials for NRR

**Figure 10:**
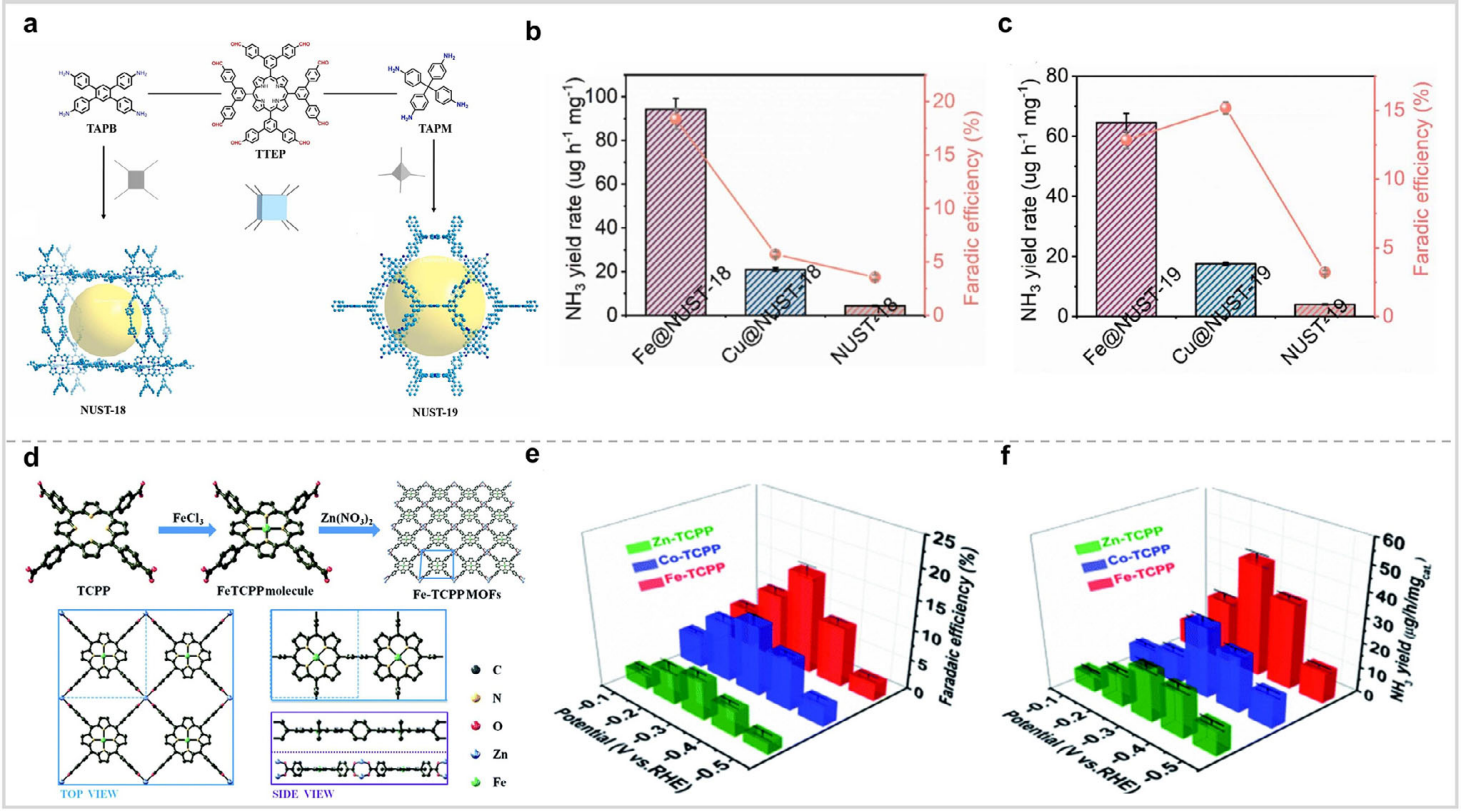
(a) Schematic synthetic process of NUST‐18 (*scu* topology) and NUST‐19 (*flu* topology). (b, c) NH_3_ yield rates and FEs of M@NUST‐18/19. Reproduced with permission.^[^ ^8e^ ^]^ Copyright 2024, Elsevier. (d) Schematic synthesis of the Fe‐TCPP MOFs. (e) FEs and (f) NH_3_ yields of M‐TCPP MOF for NRR. Reproduced with permission.^[^ ^72^ ^]^ Copyright 2021, Royal Society of Chemistry.

Por‐based derivatives not only inherit the advantages of molecular catalysts but also effectively avoid the disadvantages of molecular catalysts' aggregation. Therefore, Por‐based derivatives are an ideal material for NRR. Zhang et al. successfully synthesized Por‐COFs exhibiting unique three‐dimensional spatial configurations, which they have designated as NUST‐18 and NUST‐19, respectively (**Figure** **10**a).^[^ ^8e^ ^]^ The BET surface areas of NUST‐18 and NUST‐19 were 276 and 188 m^2^ g^−1^, respectively. They anchored Fe and Cu in Por‐COFs as catalytic sites, and their catalytic activity for NRR was tested in neutral solution. Fe@NUST‐18 had the best catalytic activity, and the maximum values of NH_3_ yield rate and FE were 94.26 ± 4.9 µg h^−1^ mg^−1^ and 18.37 ± 0.96%, respectively. The test results showed that Fe‐modified Por‐based COF had better catalytic activity than Cu‐modified Por‐based COF for NRR. This may be because Fe can activate N_2_ better than Cu. The superior catalytic performance of Fe@NUST‐18 compared to Fe@NUST‐19 can be attributed to its larger pore size and BET surface area. Like Por‐COFs, Por‐MOFs also have a regular structure and evenly dispersed active sites. Therefore, Por‐MOFs are also excellent electrocatalytic materials. Zhang et al. synthesized M‐TCPP MOF with a 2D structure by reacting MTCPP (M = Fe, Co, Zn) with Zn(NO_3_)_2_ (Figure 10d).^[^ ^72^ ^]^ The catalytic activity for NRR in a neutral electrolyte follows the descending order: Fe‐TCPP MOF > Co‐TCPP MOF > Zn‐TCPP MOF > carbon paper (CP). The peak NH_3_ yield and FE of Fe‐TCPP MOF reached 44.77 mg h^−1^ mg^−1^_cat_ and 16.23%, respectively, which are significantly larger than those of CP. This indicates that MOF is the main catalytic site, and the main role of CP is to load MOF and accelerate charge transfer. In order to better elucidate the catalytic mechanism, they carried out calculations from several perspectives. The calculation results show that Fe‐TCPP MOF activates N_2_ better than Co‐TCPP MOF, whereas Zn‐TCPP MOF does not activate N_2._ Theoretical calculations and experimental data reveal that the structural attributes and the metal center of a catalyst play a crucial role in determining the adsorption strength for reaction substrates and intermediates, which in turn influences the overall catalytic performance. Consequently, the regulation of catalyst structure and active centers remains a pivotal approach to enhance the catalytic activity of catalysts for electrocatalytic nitrogen fixation.

### 3.4 Phthalocyanine Molecules for NRR

**Figure 11:**
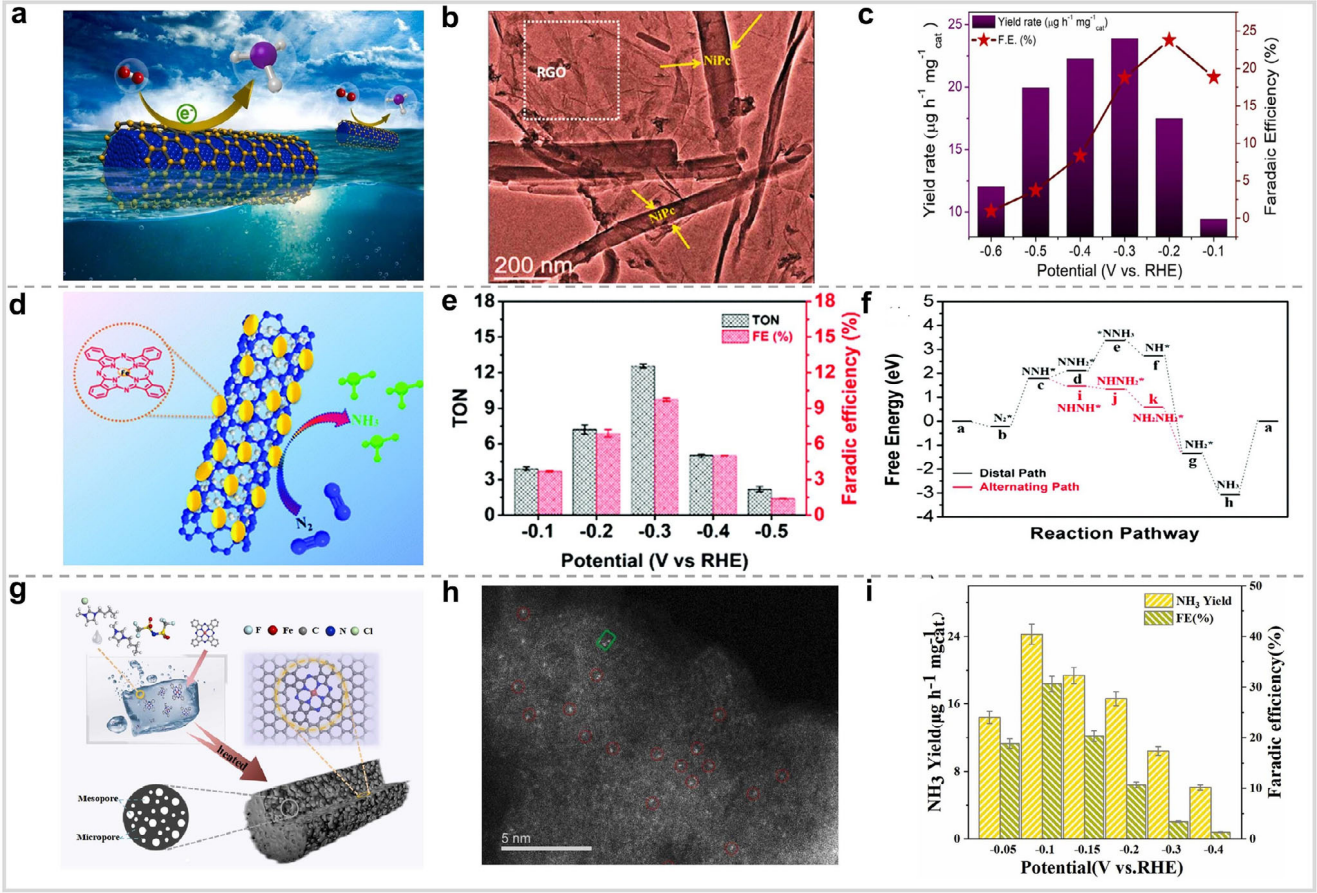
(a) Schematic diagram of NiPc‐RGO for NRR. (b) TEM image of NiPc‐RGO. (c) NH_3_ FEs and yields of NiPc‐RGO. Reproduced with permission.^[^ ^73^ ^]^ Copyright 2023, Elsevier. (d) FePc/O‐MWCNT schematic diagram for NRR. (e) The TON and NH_3_ FE of FePc/O‐MWCNT. (f) Free energy diagrams of FePc/O‐MWCNT for NRR. Reproduced with permission.^[^ ^74^ ^]^ Copyright 2019, Royal Society of Chemistry. (g) Structure and preparation process of FePc/C. (h) HAADF‐STEM images of FePc/C‐700. (i) NH_3_ yields and FEs of FePc/C‐700 at various potentials. Reproduced with permission.^[^ ^75^ ^]^ Copyright 2024, Elsevier.

Por‐based materials have shown excellent catalytic activity for NRR. Pc is similar to Por in that both are macrocyclic molecules with the ability to anchor metals to the center. Therefore, MPcs may also have excellent catalytic activity for NRR. Ghorai and colleagues investigated the catalytic activity of a composite material consisting of NiPc and reduced graphite oxide (rGo) for NRR in an acid electrolyte (**Figure** **11**a‐c).^[^ ^73^ ^]^ NiPc and NiPc‐RGO composites were characterized by FT‐IR. The test results showed that NiPc and RGO were combined via π–π interactions. The electrochemical test results show that the peak yield and FE of NiPc‐RGO reached 23.9 µg h^−1^ mg^−1^_cat_ and 23.8%. After 6 cycles, the NH_3_ yield and FE showed no significant fluctuations, indicating that catalyst maintained robust stability. The double layer capacitance (Cdl) of NiPc–RGO is quantified at 15.9 mF cm^−2^, which is markedly greater than the 0.2 mF cm^−2^ observed for NiPc. The incorporation of rGo facilitates the exposure of additional active sites on NiPc, thereby enhancing the catalytic reaction. The above studies show that Pc molecules have great development potential for NRR. Because MPc, as an organic metal complex, also has poor conductivity, it is necessary to improve the conductivity by combining with a highly conductive carrier. Cao et al. adsorbed FePc onto the surface of conductive oxidized multiwalled carbon nanotubes (O‐MWCNT) by simple adsorption (Figure 11d–f).^[^ ^74^ ^]^ The catalytic performance of FePc/O‐MWCNTs in NRR was markedly superior to that of both FePc and O‐MWCNTs individually. The peak FE and turnover number (TON) of FePc/O‐MWCNTs reached 9.73% and 12.56, respectively. Moreover, the researchers explored how altering the proportion of FePc relative to O‐MWCNTs affects the catalytic performance. When the FePc to O‐MWCNTs ratio was maintained at 7:1, the FePc/O‐MWCNTs demonstrated optimal catalytic performance. They calculated the free energy for the electrocatalytic N_2_ reduction process over FePc to investigate the reaction mechanism. Since the specific path of FePc in NRR cannot be determined, they calculated the free energy of FePc in the distal pathway and the alternate pathway, respectively. The calculation results (Figure 11f) show that FePc is more inclined to follow the alternate pathway. This is because FePc has a lower energy barrier in the alternate pathway than that of CoPc. The rate‐determining step was N_2_ →*N_2_H, with a ΔG value of 1.79 eV. This also proves that highly conductive carriers can improve the catalytic activity, and theoretical calculations are indispensable for studying the reaction mechanisms. However, due to the π–π stacking effect between the planar Pc molecules, the catalyst eventually aggregates. The aggregation of catalysts results in a reduction of exposed active sites, consequently leading to diminished catalytic activity. Zhao et al. used ionic liquid as solvent and utilized the ability of ionic liquid to oxidize metal to fully disperse FePc (Figure 11g–i).^[^ ^75^ ^]^ The uniformly mixed ionic liquid and FePc were calcined at a high temperature in a tube furnace to form Fe─N─C. The authors assert that the material, featuring a porous architecture and consistently distributed active sites, can effectively interact with the reaction substrate. The peak NH_3_ yield and FE of FePc/C‐700 were 24.25 µg h^−1^mg^−1^_cat_ and 30.59%. Theoretical simulations show that monolayer FePc is conducive to the adsorption of nitrogen. The results presented above indicate that employing ionic liquids to facilitate the dispersion of MPor, followed by calcination, constitutes an effective strategy to prevent aggregation.

### 3.5 Phthalocyanine‐Derived Materials for NRR

**Figure 12:**
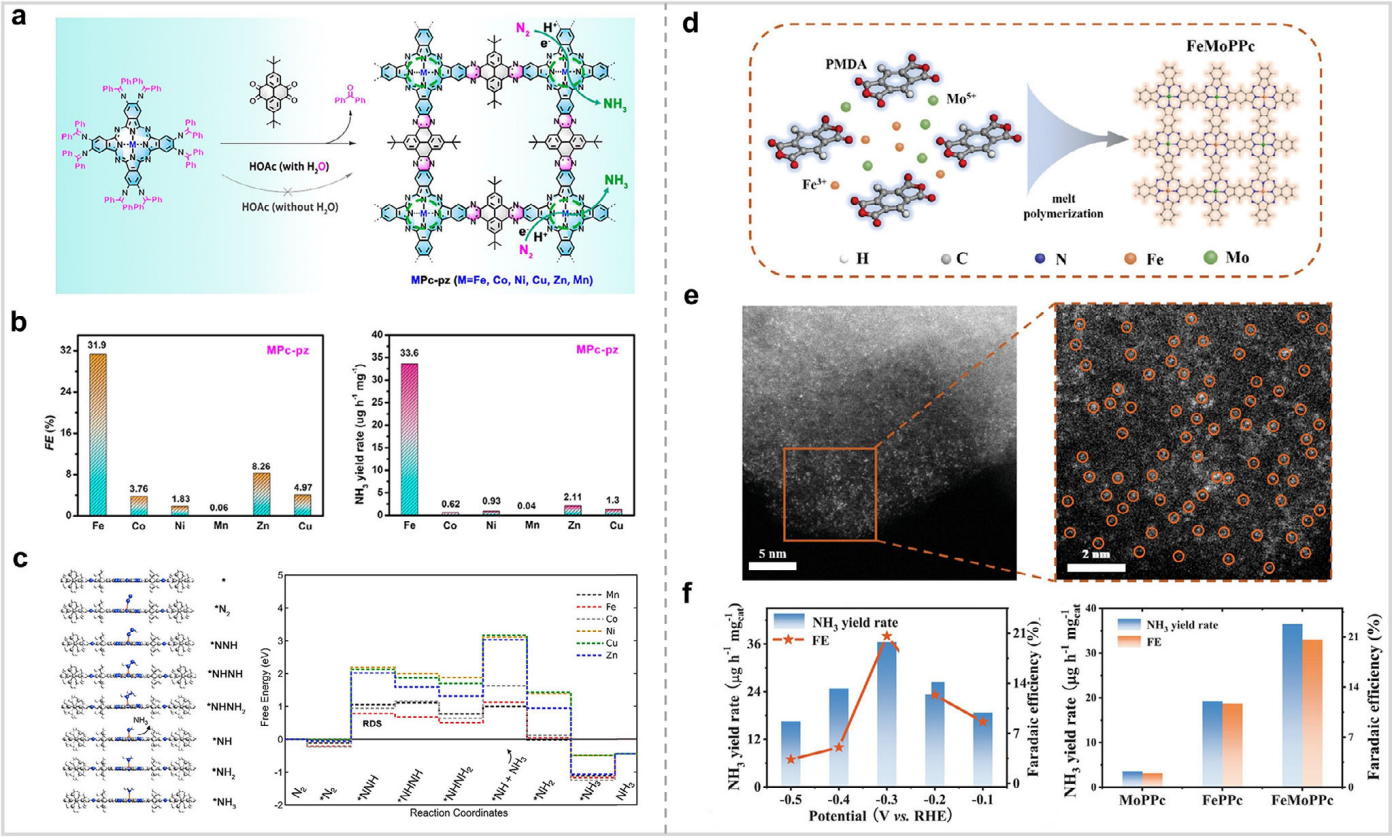
(a) Schematic synthetic process of MPc‐pz. (b) NH_3_ FEs and yield rates of MPc‐pz at −0.1 V versus RHE. (c) Optimized structures and Free energy profiles of MPc‐pz for NRR. Reproduced with permission.^[^ ^76^ ^]^ Copyright 2021, American Chemical Society. (d) Synthetic route of FeMoPPc. (e) HAADF‐STEM image of FeMoPPc. (f) NH_3_ yield rates and FEs of FeMoPPc (left), and NH_3_ yield rates and FEs of MoPPc, FePPc, and FeMoPPc (right) at −0.3 V versus RHE, respectively. Reproduced with permission.^[^ ^77^ ^]^ Copyright 2021, Wiley‐VCH GmbH.

In addition to stripping the aggregated Pc by ionic liquid, constructing Pc into COF is also an effective way to prevent Pc aggregation. Feng et al. connected MPc (M = Ni, Co, Zn, Mn, Fe, and Cu) through pyrazine to form a MPc‐based COF (MPc‐pz) with a two‐dimensional structure. (**Figure** **12**a–c).^[^ ^76^ ^]^ The electrocatalytic performance of MPc‐pz for NRR was evaluated under acidic conditions, with a focus on examining the impact of the metal center on catalytic efficacy. Among all COFs, FePc‐pz has the best catalytic performance, and its peak NH_3_ yield and FE reached 33.6 µg h^−1^ mg^−1^_cat_ and 31.9%. The catalytic behavior of FePc‐pz for the NRR was further studied using theoretical calculations. Firstly, the catalytic sites were determined by calculating the adsorption energies of N_2_ at different positions in FePc‐pz. The calculation results show that M‐N_4_‐C centers are the best N_2_ adsorption sites because of the lowest adsorption energy for N_2_. Further, the charge density difference of MPc‐pz with N_2_ adsorbed on M‐N_4_‐C centers reveals that the electron depletion region is mainly concentrated in the MN_4_ site, indicating that this site plays an important role in activating N_2_. By comparing the Gibbs free energies of N_2_ adsorption on different M‐N_4_‐C centers, it was found that Co (−0.35 eV) and Fe sites (−0.29 eV) are more likely to react with N_2_. The authors used Bader charge analysis to study the amount of charge transferred from the metal center to N_2_. The more charge that is transferred, the stronger the interaction between the catalytic site and N_2_, and the more favorable the reaction. Bader charge analysis showed that the order of the transferred charge amounts from MPc‐pz to N_2_ was Co > Fe > Mn > Ni = Zn > Cu. They also found that Fe‐N_4_‐C and Co‐N_4_‐C had the shortest M─N bond lengths between the metal and N_2_ among all M‐N_4_‐C sites, while the average M─N bond length change in their M–N_4_–C was the largest owing to the uplift heights of Fe and Co atoms. The above calculation results show that FePc‐pz and CoPc‐pz are more conducive to the adsorption and activation of N_2_ than the other MPc‐pz. The electronic structures of MPc‐pz were simulated in the presence of N_2_. The projected density of states (PDOS) for FePc‐pz indicates that the localized electronic states at the Fermi level are mainly derived from the d orbitals of metal atoms. Additionally, the electronic states associated with the N_2_ molecule engage in hybridization with the Fe orbitals located near the Fermi energy level. This further confirms that FePc‐pz is more suitable as an NRR electrocatalyst. Next, they calculated the free energy diagram of MPc‐pz in NRR as other studies. The results show that FePc‐pz has a smaller energy barrier in the alternating pathway. In summary, they confirmed the MPc‐pz site and demonstrated why FePc‐pz is more susceptible to nitrogen adsorption/activation through extensive theoretical calculations. This is important for understanding the reaction mechanism of the catalyst. Pc‐based COFs with a single metal site have shown excellent catalytic activity for NRR. COFs with dual metal sites exhibited better catalytic activity than single‐metal‐site COFs in other reactions. Therefore, Zhang et al. synthesized a Pc‐based polymer with Fe and Mo bimetallic sites (FeMoPPc) and tested its catalytic activity for NRR (Figure 12d–f).^[^ ^77^ ^]^ The electrocatalytic results showed that FeMoPPc exhibited better catalytic activity than those of FePPc and MoPPc. The NH_3_ yield of FeMoPPc was 2 and 17.2 times higher than those of FePPc and MoPPc, respectively. Theoretical studies have shown that NRR tends to occur on Fe sites rather than Mo sites. The catalytic performance of bimetallic covalent organic frameworks (COFs) surpasses that of monometallic COFs, attributable to the tandem or synergistic interactions between the two metal sites (**Table** **1**).

**Table 1:** Catalytic performance of Por/Pc‐ materials for NRR.

Catalysts	NH_3_ yield [µg h^−1^mg^−1^_cat]_	FE [%]	Electrolyte	Refs.
FeTPPCl	18.28	16.76	0.1 M Na_2_SO_4_‐PBS	[69]
CoTPP	15.18	11.43	0.1 M HCl	[70]
CuTPP	10.53	14.97	0.1 M HCl	[70]
MnTPP	7.98	8.21	0.1 M HCl	[70]
Co‐TPP‐TEG	1.10	37.00	0.1 M HCl	[71]
Fe@NUST‐18	94.26	18.37	0.1 M Na_2_SO_4_	[8e]
Cu@NUST‐18	20.94	5.72	0.1 M Na_2_SO_4_	[8e]
Fe@NUST‐19	64.49	12.85	0.1 M Na_2_SO_4_	[8e]
Cu@NUST‐19	17.55	15.18	0.1 M Na_2_SO_4_	[8e]
Fe–TCPP MOFs	44.77	16.23	0.1 M HCl	[72]
Co–TCPP MOFs	28.30	11.58	0.1 M HCl	[72]
Zn–TCPP MOFs	19.59	6.37	0.1 M HCl	[72]
NiPc–RGO	23.90	18.80	0.1 M HCl	[73]
FePc/O‐MWCNT	36	9.73	0.1 M HCl	[74]
FePc/C‐700	24.25	30.59	0.1 M HCl	[75]
FePc‐pz	33.60	31.90	0.01 M H_2_SO_4_	[76]
CoPc‐pz	3.76	0.62	0.01 M H_2_SO_4_	[76]
NiPc‐pz	1.83	0.93	0.01 M H_2_SO_4_	[76]
MnPc‐pz	0.06	0.04	0.01 M H_2_SO_4_	[76]
ZnPc‐pz	8.26	2.11	0.01 M H_2_SO_4_	[76]
CuPc‐pz	4.97	1.30	0.01 M H_2_SO_4_	[76]
FeMoPPc	36.33	20.62	0.1 M KOH	[77]
Ti‐COF	26.98	34.62	0.05 M HCl	[78]
Cu‐COF	∼9.20	∼12.50	0.05 M HCl	[78]
Co‐COF	∼10.00	∼1.00	0.05 M HCl	[78]
CoPc‐C_3_N_4_	423.80	33.00	0.1 M HCl	[79]

## 4 Nitrate/Nitrite Reduction Reaction (NO_x_RR)

Due to excessive human intervention in nature, a series of ecological and environmental problems have arisen. Excessive NO_x_^−^ in the Earth's crust is one of many questions. NO_3_^−^ and NO_2_^−^ have become common water pollutants.^[^ ^80^ ^]^ This not only pollutes the environment but also causes irreversible damage to the body. Consequently, it is imperative to mitigate or eliminate NO_x_^−^ from aquatic environments. Currently, the processes for treating nitrogen oxides in water include physical, chemical, and biological treatments.^[^ ^81^ ^]^ Physical techniques such as ion exchange and electrodialysis are available for water purification; however, they are associated with high costs, and NO_x_^−^ remains unaltered into other compounds. Industrial biological denitrification technology is characterized by significant energy consumption and instability within its reaction systems.^[^ ^82^ ^]^ In recent years, electrocatalytic technology has emerged as a highly promising field. N has various valence states, such as +5, +4, +3, +2, +1, 0, −2, and −3. Therefore, the electrocatalytic reduction of NO_3_^−^ produces various products, such as hydrazine hydrate, hydroxylamine, and NH_3_ etc. At present, the main product of NO_x_RR is NH_3_. The process of electrocatalytically reducing NO_3_^−^ to NH_3_ achieves the dual aims of purifying water and generating NH_3_.^[^ ^83^ ^]^

### 4.1 NO_x_RR reaction mechanism

**Figure 13:**
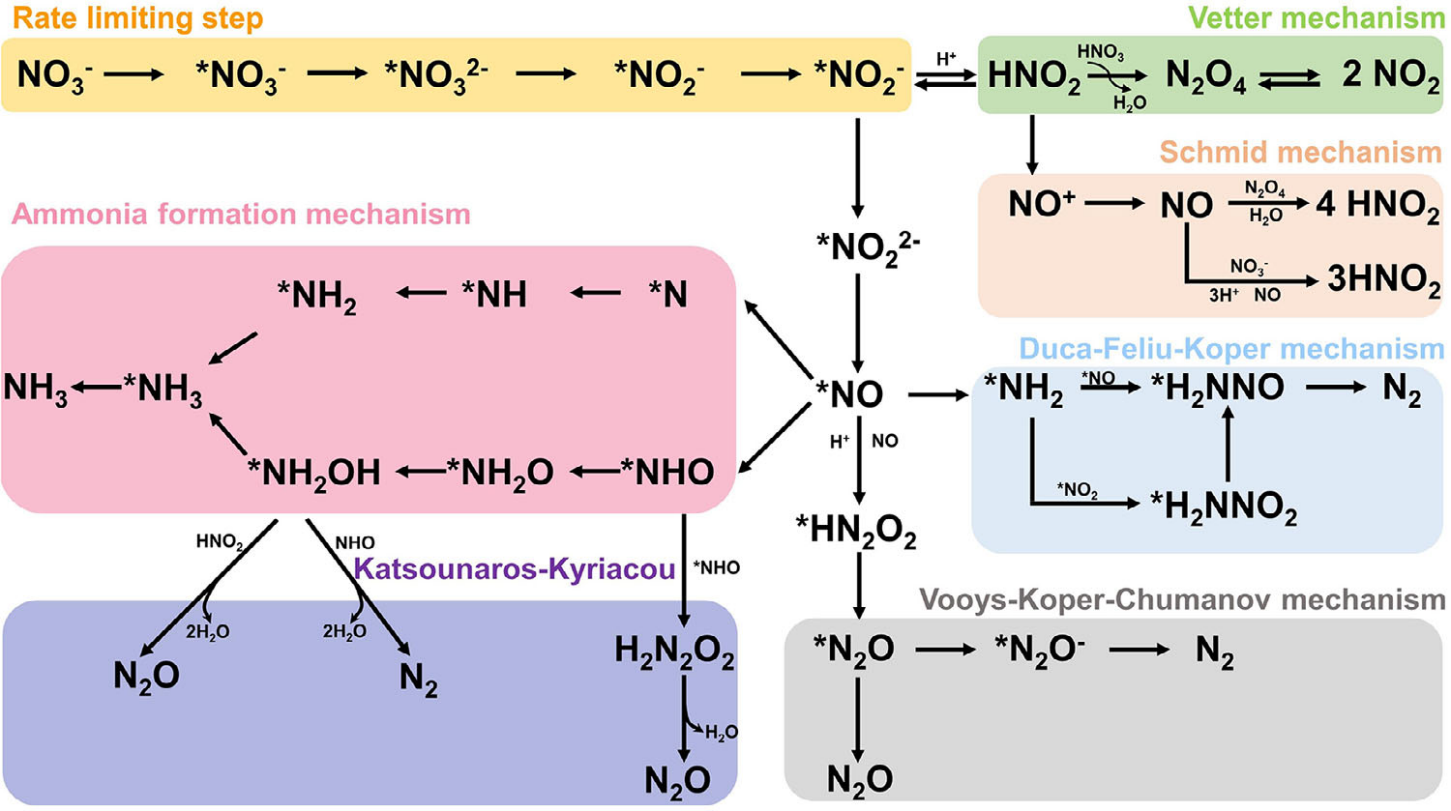
An overview of NO_3_RR mechanism in aqueous media.

During the NO_x_RR process, various products emerge simultaneously, and numerous complex intermediates are formed, resulting in diverse reaction pathways. Therefore, it is crucial to acquire a comprehensive understanding of the NO_x_^−^ reduction pathway and its underlying mechanisms before designing a catalyst.^[^ ^84^ ^]^ Recent studies suggest minor deviations in the NO_x_RR reaction pathway. However, due to the limitations of existing technology, definitive evidence to confirm the reaction pathway is lacking, leading to ongoing debate regarding the precise nature of the reaction pathway.^[^ ^4^, ^85^ ^]^ **Figure** **13** summarized the pathway for NO_3_RR.^[^ ^86^ ^]^ The reduction of NO_3_^−^ generally goes through three steps: adsorption, reduction and desorption. During the reaction, the NO_3_^−^ will be captured by the catalyst, leading to the formation of *NO_3_^−^ (* denotes the adsorbed state). *NO_3_^−^ gains two protons/two electrons and loses a water molecule to become *NO_2_^−^.^[^ ^87^ ^]^ Subsequently, due to different reaction conditions, *NO_2_^−^ will undergo different pathways. One is that in the presence of strong acid and 1.0 ∼ 4.0 M NO_3_^−^, the *NO_2_^−^ through the Vetter and Schmid routes to generate NO_2_ and NHO_2_.^[^ ^88^ ^]^ The other is that *NO_2_^−^ is further reduced to generate *NO, and then a subsequent reduction reaction occurs. *NO serves as an essential intermediate in the formation of N_2_ and NH_3_. N_2_ is generated mainly through the Vooys‐Koper mechanism^[^ ^89^ ^]^ and the Duca‐Feliu‐Koper mechanism.^[^ ^90^ ^]^ In the Duca‐Feliu‐Koper mechanism, *NO gains four protons/ four electrons and loses a water molecule to form *NH_2_. *NH_2_ continues to react with *NO or *NO_2_ to produce NH_2_NO. Finally, the unstable NH_2_NO decomposes into N_2_ and water.^[^ ^91^ ^]^ In the Vooys‐Koper mechanism, *NO takes a proton, an electron, and a NO to form *HN_2_O_2_. Eventually, this unstable *HN_2_O_2_ generates N_2_ or N_2_O.^[^ ^89^ ^]^ There are currently two different pathways for the formation of NH_3_. One is that *NO acquires two protons and two electrons and loses a water molecule to form *N, and *N is continuously hydrogenated to finally generate NH_3_ (*NO → *N → *NH → *NH_2_ → *NH_3_). Li et al. detected the signals of intermediates such as *NH, NH_2_ and *NH_3_ using in situ FT‐IR.^[^ ^92^ ^]^ Guo et al. further confirmed this response pathway.^[^ ^93^ ^]^ Another pathway is that *NO gains three electrons/three protons to generate *NH_2_OH, and *NH_2_OH gains two protons, two electrons, and releases a water molecule to generate NH_3_ (*NO → *NHO → *NH_2_O → *NH_2_OH → *NH_3_). Zhang's team detected the presence of NH_2_OH through DEMS, proving that the formation of NH_3_ is related to NH_2_OH.^[^ ^94^ ^]^ Sun and co‐workers detected the presence of NH_2_OH by in situ FT‐IR.^[^ ^95^ ^]^ The above research shows that in‐situ technology provides a strong guarantee for studying the reaction mechanism of NO_3_RR.

### 4.2 Porphyrin molecules for NO_x_RR

**Figure 14:**
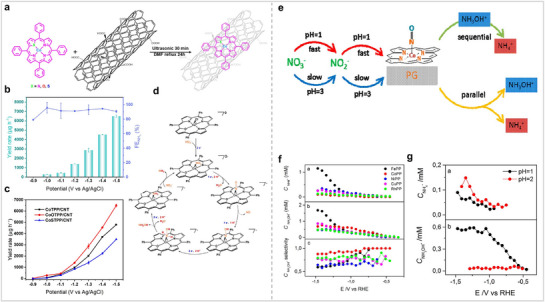
(a) The structures of CoTPP (X═N), CoOTPP (X═O), CoSTPP (X═S). (b) NH_3_ yield rate and FE of CoOTPP/CNT. (c) NH_3_ yield rates of CoTPP/CNT, CoOTPP/CNT, and CoSTPP/CNT. (d) Reaction mechanism for NO_2_RR via CoOTPP. Reproduced with permission.^[^ ^97^ ^]^ Copyright 2024, Wiley‐VCH GmbH. (e) The reaction path of NO_3_RR by CoPor immobilized on a pyrolytic graphite. (f) Concentration of NH_4_^+^and NH_3_OH^+^, and selectivity of NH_3_OH^+^. (g) NH_4_^+^and NH_3_OH^+^ concentration of CoPor‐immobilized PG electrode. Reproduced with permission.^[^ ^98^ ^]^ Copyright 2015, American Chemical Society.

In 1986, Meyer's group studied the catalytic activity of FePor for NO_2_RR in aqueous solution and discussed the reaction mechanism.^[^ ^96^ ^]^ The water‐soluble FePor‐SO_3_H ([Fe^III^(H_2_O)(TPPS)]^3−^) can effectively reduce NO_2_^−^ to NH_3_, NH_2_OH, and N_2_O. The reaction pathway of FePor reducing NO_2_^−^ to NH_3_ was studied by adjusting the pH of the solution. They believe that NH_2_OH is an important intermediate in the process of MPor reducing NO_2_^−^ to NH_3_. This pathway has been confirmed and recognized by researchers. Our research group has mainly studied the design of Por/Pc molecules and their electrocatalytic applications. The catalytic performance and product selectivity of MPors for electrocatalytic nitrogen fixation are subject to modification through the manipulation of their surrounding microenvironment. We synthesized three MPors with different inner ring structures (**Figure** **14**a, Co‐N_3_X_1_, X═N, O, S) and studied their ability to reduce NO_2_^−^.^[^ ^97^ ^]^ Among the three MPors, CoTOPP (Co‐N_3_O_1_) demonstrated superior catalytic performance, achieving an NH_3_ FE exceeding 90% within a potential range of 0.6 V. We studied the reaction mechanism, and the results showed that adjusting the microenvironment of MPor, the electron transfer path can be changed, thereby regulating the catalytic performance of MPor. The above studies prove that changing microenvironment of MPors is still effective in changing their activity for NO_2_RR. Koper et al. synthesized MPors (M═Ni, Rh, Fe, Cu, and Co) with different metals and fixed them on pyrolytic graphite to test their catalytic activity for NO_3_RR (Figure 14e–g).^[^ ^98^ ^]^ Among all MPors, CoPor has the highest selectivity for NH_2_OH, so the authors selected CoPor for subsequent research. The impact on the product was investigated by altering the solution pH and the potential. The experimental data revealed a strong correlation between the catalytic performance, product selectivity of CoPor, and the pH. As the pH decreased, the selectivity of NH_2_OH also increased, and the electrolysis results showed that the potential also had a great influence on the selectivity of the product. When the pH was 1, the selectivity of NH_2_OH was close to 100% at −0.5 V versus RHE, and the selectivity of NH_3_ was significantly increased at −0.75 V versus RHE. Their research demonstrated that the pH of the solution and the electrolysis potential are critical factors influencing the catalytic activity and product selectivity of the catalyst. The above studies show that MPor has great application potential for NO_3_RR.

### 4.3 Porphyrin‐Derived Materials for NO_x_RR

**Figure 15:**
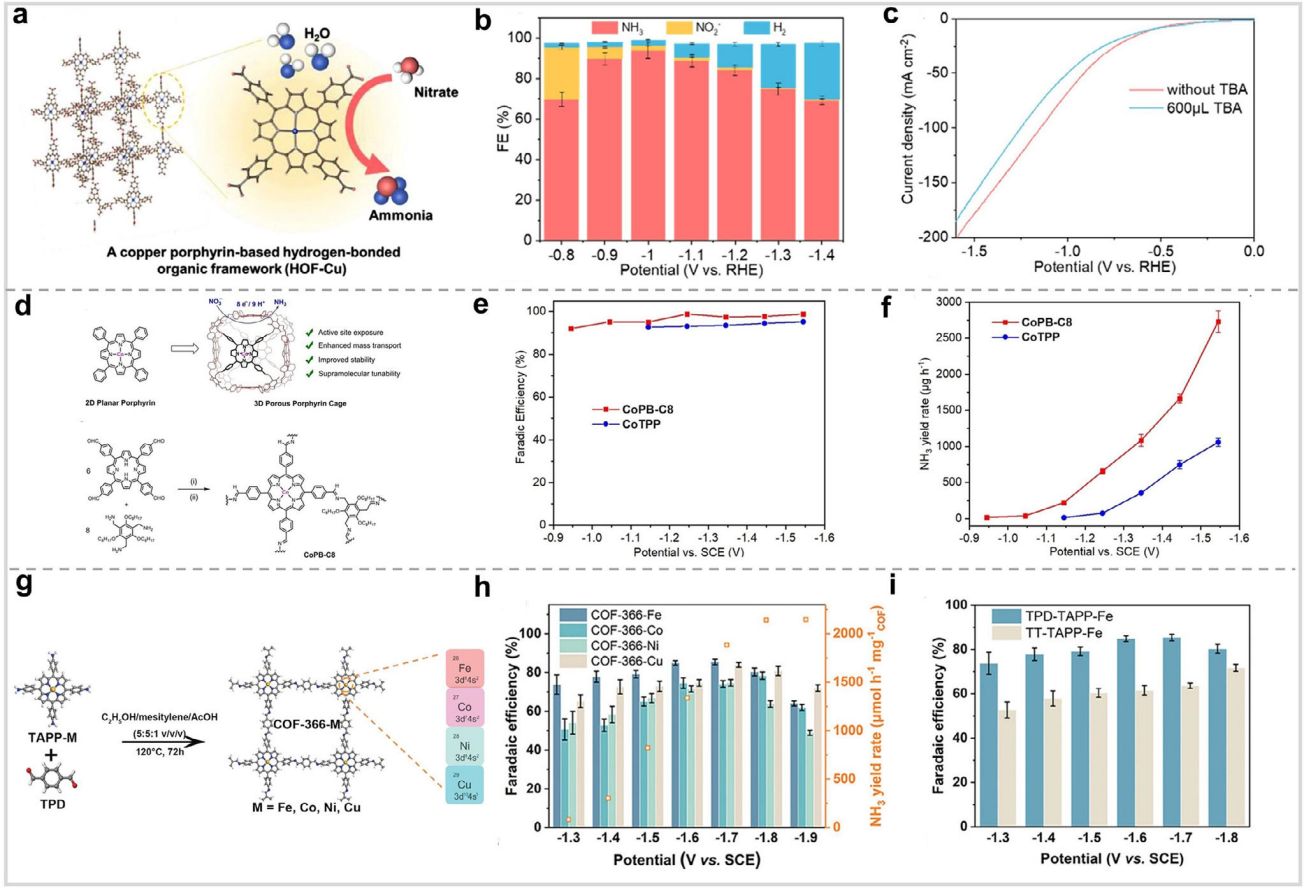
(a) Structure and catalytic diagrams of HOF‐Cu. (b) Different product FEs of HOF‐Cu for NO_3_RR. (c) LSV curves of HOF‐Cu. Reproduced with permission.^[^ ^99^ ^]^ Copyright 2024, American Chemical Society. (d) Structure diagram of MPor‐based 3D supramolecular cages. (e) NH_3_ FE of CoPB‐C8 and CoTPP during NO_3_RR. (f) NH_3_ yield rate for CoPB‐C8 and CoTPP. Reproduced with permission.^[^ ^100^ ^]^ Copyright 2023, Wiley‐VCH GmbH. (g) COF‐366‐M Synthesis Pathway. (h) NH_3_ FE and yield rate of COF‐366‐M for NO_3_RR. (i) NH_3_ FE of COF‐366‐Fe and TT‐TAPP‐Fe. Reproduced with permission.^[^ ^101^ ^]^ Copyright 2023, Wiley‐VCH GmbH.

At present, MPor‐based organic frameworks have been developed to a relatively complete level, and they have shown excellent catalytic activity in other electrocatalytic fields. This provides a good foundation for the application of MPor‐based organic frameworks in NO_3_RR. Hou et al. synthesized a CuPor‐based hydrogen‐bonded organic framework (HOF‐Cu). Compared with HOF, HOF‐Cu has better catalytic activity for NO_3_RR (**Figure** **15**a–c).^[^ ^99^ ^]^ The peak NH_3_ FE and yield were 93.8% and 0.65 mmol h^−1^ cm^−2^. After 20 cycles, the NH_3_ FE and NH_3_ yield did not decrease significantly. This shows that HOF‐Cu has good stability. To prove that *H dissociated from water participated in NO_3_RR, rather than HER, they used tert‐butanol (TBA) to quench *H. LSV showed that the current intensity decreased with the addition of TBA, indicating that *H participated in NO_3_RR. The reaction mechanism of HOF‐Cu for NO_3_RR was studied using in situ FT‐IR, DEMS, and density functional theory (DFT) calculations. The results show that the reaction pathway involves the continuous deoxidation of NO_3_^−^ to *NO and hydrogenation to NH_3_ (*NO →*HNO → *NH_2_OH → NH_3_). Chang et al. proposed a supramolecular strategy to integrate 2D CoPor units into three‐dimensional porous organic cages and successfully synthesized CoPB‐C8 Por cages (Figure 15d–f).^[^ ^100^ ^]^ For NO_3_RR, the catalytic potential of the CoPB‐C8 Por cage was positively shifted by 200 mV, and the catalyst activity increased by 15 times compared with CoPor. The Por cage enhances the exposure of the active site and promotes the interaction between the substrate and the catalyst, and the peripheral alkyl substituents have a regulatory effect on the supramolecular porosity and cavity size. Their work demonstrates that assembling MPor/Pc into 3D cages is also a good strategy to disperse MPor. Lu et al. synthesized COF‐366‐M using MPor‐NH_2_ (M = Fe, Co, Ni, or Cu) and terephthalaldehyde (TPD) as ligands (Figure 15g–i).^[^ ^101^ ^]^ The electrolysis data revealed that the catalytic performance of COF‐366‐M in NO_3_RR was ranked in the following order: Fe > Cu > Ni > Co. The maximum NH_3_ FE and yield of COF‐366‐Fe were 85.4% and 1883.6 µmol h^−1^ mg^−1^_COF._ The author substituted TPD with the thieno[3,2‐b]thiophene‐2,5‐dicarbaldehyde (TT) monomer to synthesize TT‐TAPP‐M. Compared to COF‐366‐Fe, the Fe in TT‐TAPP‐Fe exhibited a higher charge density, which consequently impaired its effectiveness in NO_3_RR. Since *NO is a key intermediate in the generation of NH_3_, the authors proposed the *NO binding energy to evaluate the ability of COF for NO_3_RR. The authors found that the higher the G (*NO), the lower the ∆G (*NO‐*NHO), which is more conducive to the reaction and is consistent with the experimental results. This study shows that after MPor is assembled into COF, changing the type of metal is still effective in regulating their catalytic activity for NO_3_RR. Hod et al. tuned the catalytic activity of FePor‐based metal–organic frameworks for NO_2_RR by installing proton relays.^[^ ^102^ ^]^ The authors first synthesized a two‐dimensional MOF (Zr‐BTB), then FePor was fixed on Zr‐BTB to synthesize Zr‐BTB@Hemin. Finally, a series of proton‐relaying sequential sphere functionalities was incorporated into Zr‐BTB@Hemin. The immobilization of pendant OH groups in Zr‐BTB@Hemin‐THBA can greatly enhance the selectivity and rate of electrocatalysis. The NH_3_ selectivity was 90%. The authors believe that the OH functional group can enhance the cleavage of the N‐O in NO_2_^−^ to generate NO, which combines with Fe and goes to generate the Fe‐NO intermediate. Fe‐NO intermediate is the key intermediate in the production of NH_3_. Previous research involved the calcination of MPor to produce M‐N‐C materials characterized by their distinct active sites. M‐N‐C materials have been identified as exemplary electrocatalytic materials. Based on previous research, Chen et al. mixed MPor and reduced graphene oxide (rGO) in a solution, and then the mixture was annealed at a high temperature to obtain Cu_x_M_1‐x_‐N‐C catalysts.^[^ ^103^ ^]^ Electrochemical tests showed that the main reduction product of Cu_x_M_1‐x_‐N‐C catalysts for NO_3_RR was N_2_. The Cu_0.8_Ni_0.2_‐N‐C achieved 74.9% total nitrogen removal, 95.0% nitrogen selectivity, and 35.5% energy utilization efficiency. The authors believe that Cu/Ni‐N‐C has a synergistic catalytic effect. The above studies show that integrating MPor into organic frameworks is an effective means to regulate catalytic activity for NO_3_RR. However, since the application of MPor‐based catalysts in NO_3_RR has only been paid attention to in recent years, related research is not perfect. Therefore, the application of MPor‐based catalysts in NO_3_RR needs to be further improved.

### 4.4 Phthalocyanine Molecules for NO_X_RR

**Figure 16:**
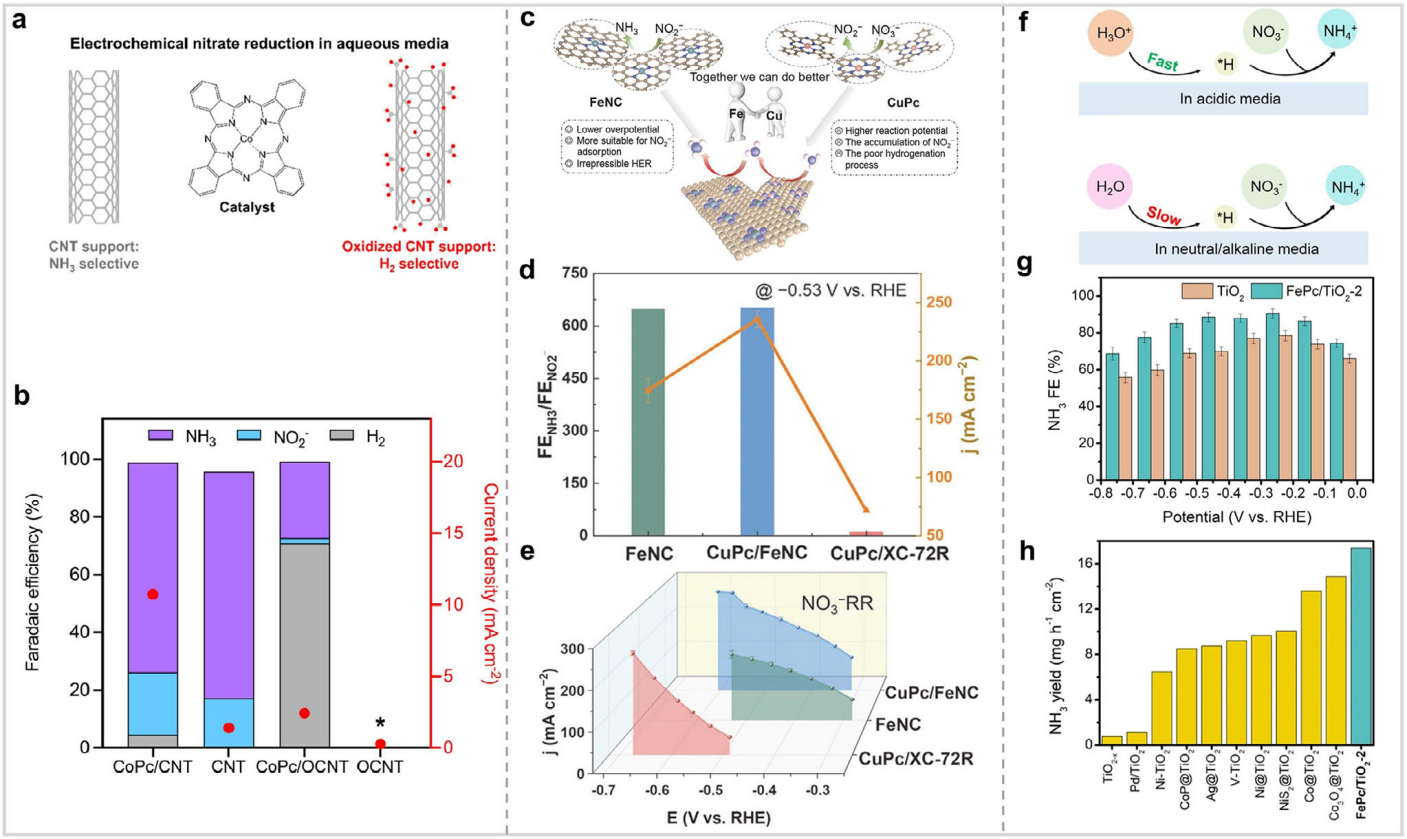
(a) Schematic diagram of CoPc and carrier. (b) Current density and product distribution of catalysts at −0.37 V versus RHE. Reproduced with permission.^[^ ^105^ ^]^ Copyright 2024, American Chemical Society. (c) Tandem catalytic mechanism of CuPc/FeNC for NO_3_RR. (d) FE NH_3_/FE NO_2_^−^ and NH_3_ partial current density. (e) NH_3_ partial current density. Reproduced with permission.^[^ ^106^ ^]^ Copyright 2024, Elsevier. (f) Sources pathways of *H for NO_3_RR in different electrolytes. (g) NH_3_ FE of TiO_2_ and FePc/TiO_2_‐2. (h) Comparison of NH_3_ FE for NO_3_RR. Reproduced with permission.^[^ ^107^ ^]^ Copyright 2023, Springer Nature.

MPcs have been proven to be an effective electrocatalyst for NO_2_RR and NO_3_RR. Nyokong and their research team conducted an investigation into the catalytic properties of MPcs containing various metals, such as Mn, Fe, Co, Ni, Cu, and Zn, for NOxRR in alkaline environments.^[^ ^104^ ^]^ The main product of MPcs reduction of NO_2_^−^ or NO_3_^−^ was NH_3_. CuPc showed the best reduction ability for NO_2_^−^ or NO_3_^−^ among all MPcs. MPcs have different catalytic activity for NO_3_RR because the adsorption, desorption, and conversion capabilities of MPcs for substrates show significant differences during the electrocatalytic process, which ultimately results in different catalytic activities. The catalyst carrier is also an important factor affecting the catalytic performance. The catalytic performance of Por/Pc‐based catalysts, which inherently exhibit poor conductivity, can be markedly enhanced by supporting them on a substrate that possesses either superior conductivity or catalytic activity. Therefore, Wang et al. loaded CoPc onto CNTs with different properties and studied their catalytic activities for NO_3_RR (**Figure** **16**a).^[^ ^105^ ^]^ With ordinary carbon nanotubes (CNT) as carriers, CoPc/CNT can efficiently reduce NO_3_^−^ to NH_3_, and the NH_3_ FE can reach 70%. With oxidized carbon nanotubes (OCNT) as carriers, the reduction product of CoPc/OCNT was mainly H_2_, only part of the NO_3_^−^ was reduced to NH_3_, and the NH_3_ FE was only 27%. The authors believe that the oxygen functional groups on OCNTs facilitate proton transfer, so the main reduction product of CoPc is H_2_. Wang et al. synthesized the catalyst (CuPc/FeNC) with Fe and Cu sites by combining N‐coordinated Fe single‐atoms with CuPc (Figure 16c).^[^ ^106^ ^]^ Compared with the single‐component catalyst, CuPc/FeNC exhibits a larger current intensity and a more positive catalytic potential. The maximum NH_3_ FE was close to 100% and the current density corresponding to the production of NH_3_ reached 273 mA cm^−2^. They believe that the Cu and Fe sites play a tandem catalytic role, which was verified through experiments and theoretical calculations. The main function of Cu is to reduce NO_3_^−^, while the main function of Fe is to convert NO_2_^−^ produced by Cu. Research findings indicate that the presence of catalyst carriers plays a crucial role in modulating the catalytic efficiency. Therefore, the choice of carrier is very important. In catalytic reactions, due to the difference in the ability of the electrolyte to provide protons, this will also have an effect on the catalytic activity of the catalyst. Most of the reported for NO_x_RR is in neutral or alkaline media, but there are few reports in acidic media. Zhi et al. synthesized FePc/TiO_2_ by hybridization of TiO_2_ and FePc (Figure 16f–h).^[^ ^107^ ^]^ Electrochemical testing reveals that NH_3_ is the predominant reduction product of FePc/TiO2‐2 during NO_3_RR. The NH_3_ yield can reach 17.4 mg h^−1^ cm^−2^ and the maximum NH_3_ FE reached 90.6%. The authors assert that the integration of FePc with TiO_2_ enhances the adsorption capacity for NO_3_^−^, thereby substantially increasing the catalyst's effectiveness for NO_3_RR. Simultaneously, there is an abundant proton supply and fast kinetics of NO_3_RR under acidic conditions. The above studies show that MPc‐based catalysts have shown excellent catalytic activity, and NH_3_ FE can be close to 100%. At the same time, the influence of carrier and solution on catalytic activity cannot be ignored. Therefore, in addition to the directional synthesis of MPc‐based catalysts, appropriate carriers and solutions must be selected.

### 4.5 Phthalocyanine‐Derived Materials for NO_x_RR

**Figure 17:**
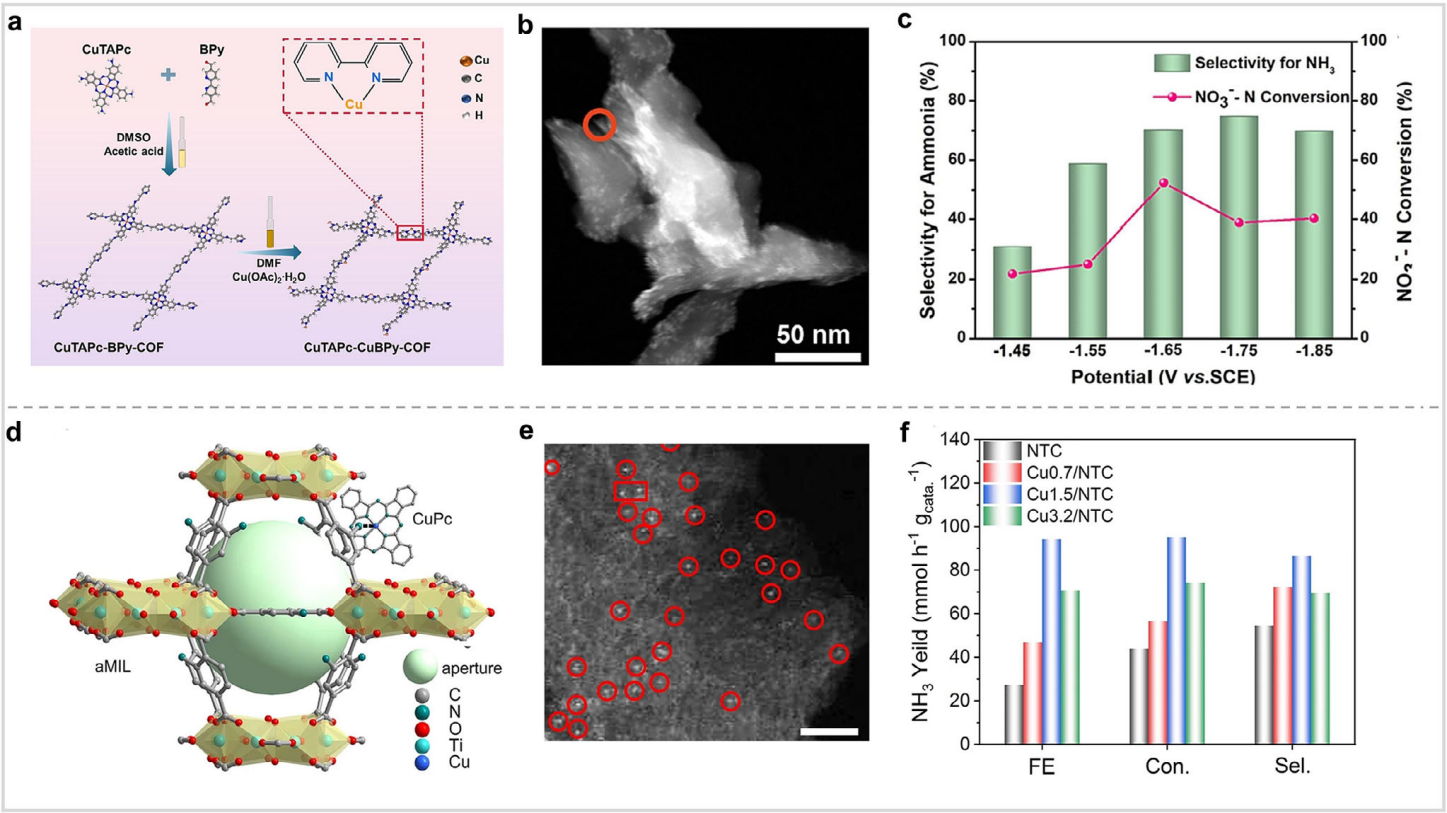
(a) Schematic diagram of catalyst synthesis. (b) HRTEM of CuTAPc‐CuBPy‐COF. (c) NH_3_ selectivity and NO_3_^−^ conversion of CuTAPc‐CuBPy‐COF. Reproduced with permission.^[^ ^108^ ^]^ Copyright 2024, Elsevier. (d) Schematic diagram of aMIL‐CuPc. (e) HAADF‐STEM image of Cu_0.7_/NTC. (f) Catalytic performance comparison of Cu_x_/NTC for NO_3_RR at −0.75 V versus RHE. Reproduced with permission.^[^ ^109^ ^]^ Copyright 2023, Springer Nature.

MPcs have been demonstrated to be effective electrocatalysts for NO_x_RR. Since NO_x_RR is an electron‐proton coupling reaction, the reaction process is complex and involves multiple intermediates, which makes the catalytic effect of MPcs with a single catalytic site at a medium level. Therefore, in addition to strategies such as regulating the microenvironment of MPc and selecting appropriate carriers, constructing MPc‐based catalysts with multiple catalytic sites is also a very effective strategy. Li et al. synthesized a CuTAPc‐BPy‐COF with 2D construction using CuPc as building blocks via a Schiff base reaction. Because bipyridine can trap metal ions, they synthesized CuTAPc‐CuBPy‐COF with two different Cu sites by reacting CuTAPc‐BPy‐COF with copper salts (**Figure** **17**a).^[^ ^108^ ^]^ The catalytic performance of CuTAPc‐CuBPy‐COF surpassed that of CuTAPc‐BPy‐COF, achieving a 90.3% NO_3_^−^ conversion rate. Although Cu‐based catalysts have good reactivity for NO_3_RR, but the reorganization of Cu‐based electrocatalysts during the reaction hinders the study of catalytic mechanisms. Tao et al. loaded different contents of Cu on N‐doped TiO_2_/C (Cu_x_/NTC) by anchoring different amounts of CuPc in NH_2_‐MIL125 (Ti) followed by pyrolysis strategy (Figure 17d–f).^[^ ^109^ ^]^ In the reconstruction of Cu, Cu^x+^ is reduced to Cu^0^, and then Cu^0^ migrates and aggregates to form Cu clusters. The reconstruction of the Cu was associated with both the Cu loading content and the applied voltage. The dispersion state of Cu in Cu_x_/NTC was verified by HAADF‐STEM. The Cu present in Cu_0.7_/NTC exhibited a monodisperse configuration, with the spacing between individual Cu atoms being approximately 0.72 nm. Single Cu atoms and Cu clusters were simultaneously observed in Cu_1.5_/NTC. The (111) crystal plane of Cu was observed in Cu_3.2_/NTC, indicating that Cu existed in the form of nanoparticles. The Cu_1.5_/NTC catalyst, characterized by the presence of CuN_4_ and Cu_4_, exhibits superior catalytic performance. As indicated by the data, the yield of NH_3_ was found to be 0.044 mmol h^−1^ cm^−2^, while the FE of NH_3_ was determined to be 94.3% at −0.75 V versus RHE. The calculation results show that the regulation of charge distribution and the d‐band center of the catalytic center are influenced by an appropriate number of Cu clusters, thereby enhancing the adsorption of NO_3_^−^ (**Table** **2**).

**Table 2:** Comparison of the NO_x_RR performance of porphyrin/phthalocyanine‐based materials.

Catalysts	NH_3_ Yield [µg h^−1^mg_cat_^−1^]	FE [%]	Electrolyte	Refs.
CoOTPP/CNT	32 490	94.5	0.5 M Na_2_SO_4_ 0.1 M NaNO_2_	[97]
HOF‐Cu	13 812	93.8	0.5 M K_2_SO_4_ 0.1 M KNO_3_	[99]
CoTPP/GC	/	94	0.5 M Na_2_SO_4_ 0.1 M NaNO_3_	[100]
CoPB‐C8/GC	/	98	0.5 M Na_2_SO_4_ 0.1 M NaNO_3_	[100]
COF‐366‐Fe	32 021	85.4	0.5 M K_2_SO_4_ 0.1 M KNO_3_	[101]
Zr‐BTB@Hemin	/	58	0.2 M Na_2_SO_4_ 0.1 M NaNO_2_	[102]
Zr‐BTB@Hemin‐MHBA	/	63	0.2 M Na_2_SO_4_ 0.1 M NaNO_2_	[102]
Zr‐BTB@Hemin‐DHBA	/	70	0.2 M Na_2_SO_4_ 0.1 M NaNO_2_	[102]
Zr‐BTB@Hemin‐THBA	/	83	0.2 M Na_2_SO_4_ 0.1 M NaNO_2_	[102]
CoPc/CNT	/	70	0.5 M KOH 0.04 M KNO_3_	[105]
CoPc/OCNT	/	27	0.5 M KOH 0.04 M KNO_3_	[105]
CuPc/FeCN	/	∼100	1.0 M KOH 0.2 M KNO_3_	[106]
FePc/TiO_2_‐2	33 080	90.6	0.1 M HNO_3_ 0.4 M KNO_3_	[107]
CuTAPc‐CuBPy‐COF	/	69.6	0.5 M Na_2_SO_4_ 0.007 M NO_3_^−^	[108]
Cu_1.5_/NTC	1499	94.3	0.5 M Na_2_SO_4_ 5.88×10^−4^ M NaNO_3_	[109]
CuPc@MXene	1340	/	0.5 M Na_2_SO_4_ 5.88×10^−4^ M NaNO_3_	[110]
FePc@MXene	660	/	0.5 M Na_2_SO_4_ 5.88×10^−4^ M NaNO_3_	[110]
CoPc@MXene	360	/	0.5 M Na_2_SO_4_ 5.88×10^−4^ M NaNO_3_	[110]
NiPc@MXene	600	/	0.5 M Na_2_SO_4_ 5.88×10^−4^ M NaNO_3_	[110]
NiPr	830	～85	0.5 M K_2_SO_4_ 0.1 M KNO_3_	[111]
NiPr‐TPA‐COF	2500	～90	0.5 M K_2_SO_4_ 0.1 M KNO_3_	[111]
NiPc‐CNT	/	86.8	0.05 M Na_2_SO_4_ 3.57×10^−3^ M NaNO_3_	[112]
MnPc/RGO	20 316	98.3	0.1 M K_2_SO_4_ 0.2 M KNO_3_	[113]

## 5 Electrochemical Synthesis of Urea via NO_x_/CO_2_ Co‐Reduction

Urea (NH_2_CONH_2_) is an important intermediate substance in the N cycle, and the N content in urea is 46%.^[^ ^114^ ^]^ Furthermore, urea is an essential compound for human development, serving as a critical raw material in industrial sectors. According to statistics, humans consume approximately 100 million tons of urea each year.^[^ ^115^ ^]^ Currently, urea is synthesized from NH_3_ and CO_2_ via the Bosch–Meiser process. However, the Bosch–Meiser processes are both energy‐intensive processes.^[^ ^116^ ^]^ Electrocatalysis has become a promising technology for urea synthesis because of the development of green, electrical energy. At present, synthesis of urea by electrocatalytic technology usually uses NO_x_^−^ and CO_2_, N_2_, CO_2_, NO and CO_2_, and NH_3_ and CO, etc.^[^ ^117^ ^]^

Electrocatalytic synthesis of urea from N_2_ and CO_2_ is hindered due to the stability and low solubility of N_2_. Compared with N_2_, NO_x_^−^ has high solubility in water and the N‐O bond is weaker.^[^ ^118^ ^]^ Therefore, NO_x_^−^ is an ideal raw material for synthesizing urea, which can not only purify water but also produce urea with high added value. Currently, most studies on the synthesis of urea by electrocatalytic technology use NO_x_^−^ as the N source, and only a few studies use N_2_ as the N source. As predicted by theory, both the yield and FE of synthesized urea using N_2_ as an N source were significantly lower than those of synthesized urea using NO_x_^−^ as an N source. In 1998, Furuya synthesized urea from NO_3_^−^ and CO_2_ via electrocatalytic technology.^[^ ^119^ ^]^ The catalytic activity of part transition metals was studied. Zn exhibited the best catalytic activity, and the maximum urea FE was approximately 35%. It is asserted that the catalyst's ability to effectively drive NO_3_RR and CO_2_RR is a fundamental requirement for urea synthesis. Compared with NO_3_^−^, NO_2_^−^, and CO_2_ co‐reduction is more likely to produce urea. The above research has a good inspiration for the synthesis of urea by C─N coupling through electrocatalysis technology. Over the next ten years, the research community largely overlooked the electrocatalytic synthesis of urea. However, in recent years, with the advancement of electrocatalytic and in‐situ characterization technologies, this area of research has once again attracted scholarly interest.^[^ ^120^ ^]^

### 5.1 Electrochemical Urea Synthesis Reaction Mechanism

**Figure 18:**
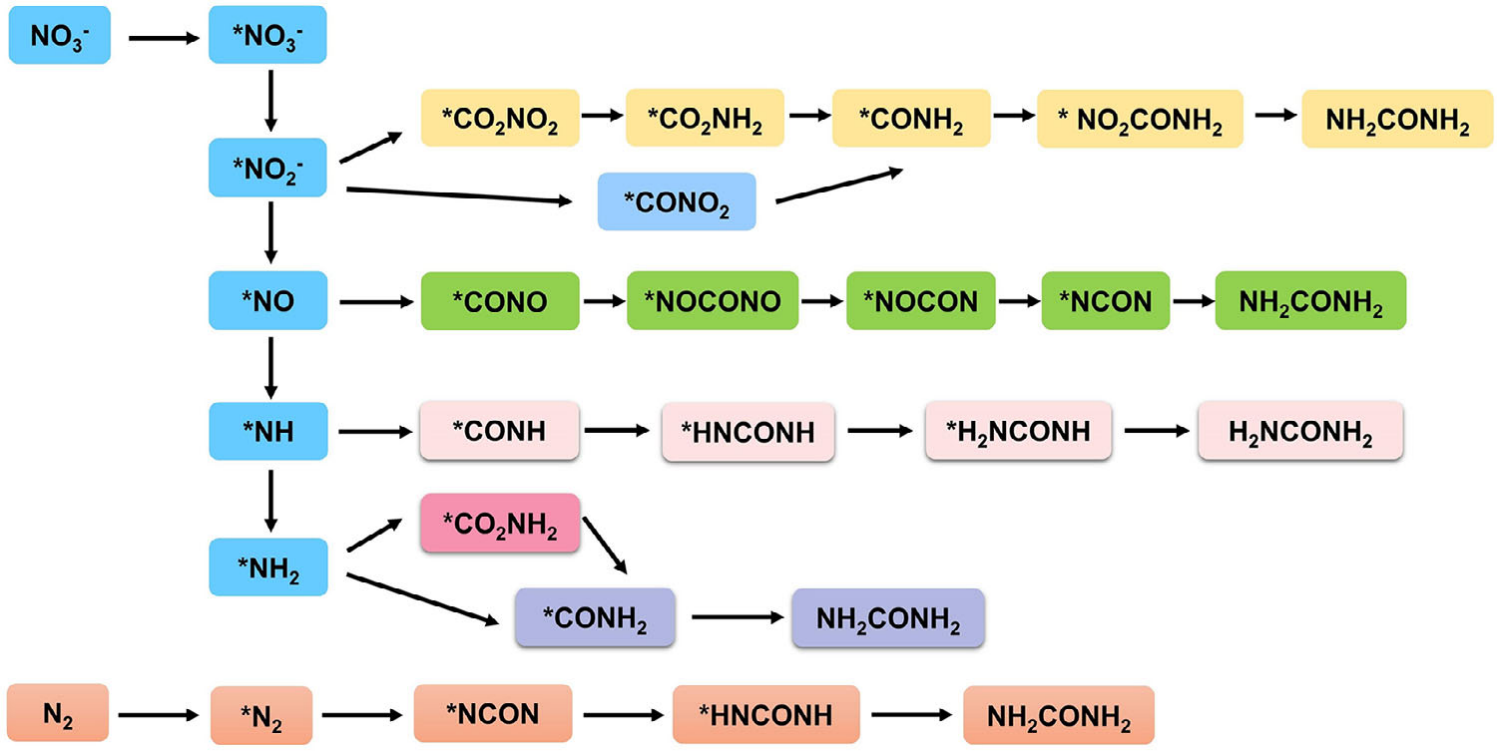
Possible reaction pathways for C─N coupling.

The process of electrocatalytic urea synthesis is intricate, involving over seventy intermediates. However, the absence of direct evidence to identify the key intermediates renders the steps and mechanism of this synthesis unclear.^[^ ^117a^ ^]^ We present a summary of the potential pathways for urea synthesis developed in recent years, as illustrated in **Figure** **18**. The identification of the principal intermediates involved in urea synthesis remains a subject of ongoing debate. The intermediates, *NO_2_, *NO, *NH_2_, *NH, and *CO, are regarded by researchers as crucial intermediates for C‐N coupling. In 2022, Qiao et al. performed a simulation through theoretical calculation to research the C─N coupling mechanism.^[^ ^121^ ^]^ Simulation results show that the C─N coupling reaction is restricted to a limited potential range, occurring exclusively at low potentials. Consequently, they investigated the primary reaction intermediates for urea synthesis on Cu (100). Experimental findings reveal that *NH and *CO are essential precursors for the formation of C─N bonds at low overpotentials. However, at high overpotentials, the C─N coupling takes place between *NH and the solvated CO. After summarizing, it was found that the most recognized mechanism by researchers was the reduction of NO_x_^−^ to *NH_2_ and CO_2_ is reduced to *CO. Then, NH_2_ and *CO are coupled to form urea. However, there is still a lack of direct evidence to support the coupling of *NH_2_ and *CO to produce urea. Therefore, the mechanism of electrocatalytic synthesis of urea needs further study.^[^ ^122^ ^]^ They studied the reaction pathway of C─N coupling by combining simulation and experiment, which provided guidance for the subsequent exploration of electrocatalytic C─N coupling reactions.

### 5.2 Porphyrin‐Based Catalysts For Electrocatalytic Synthesis of Urea

**Figure 19:**
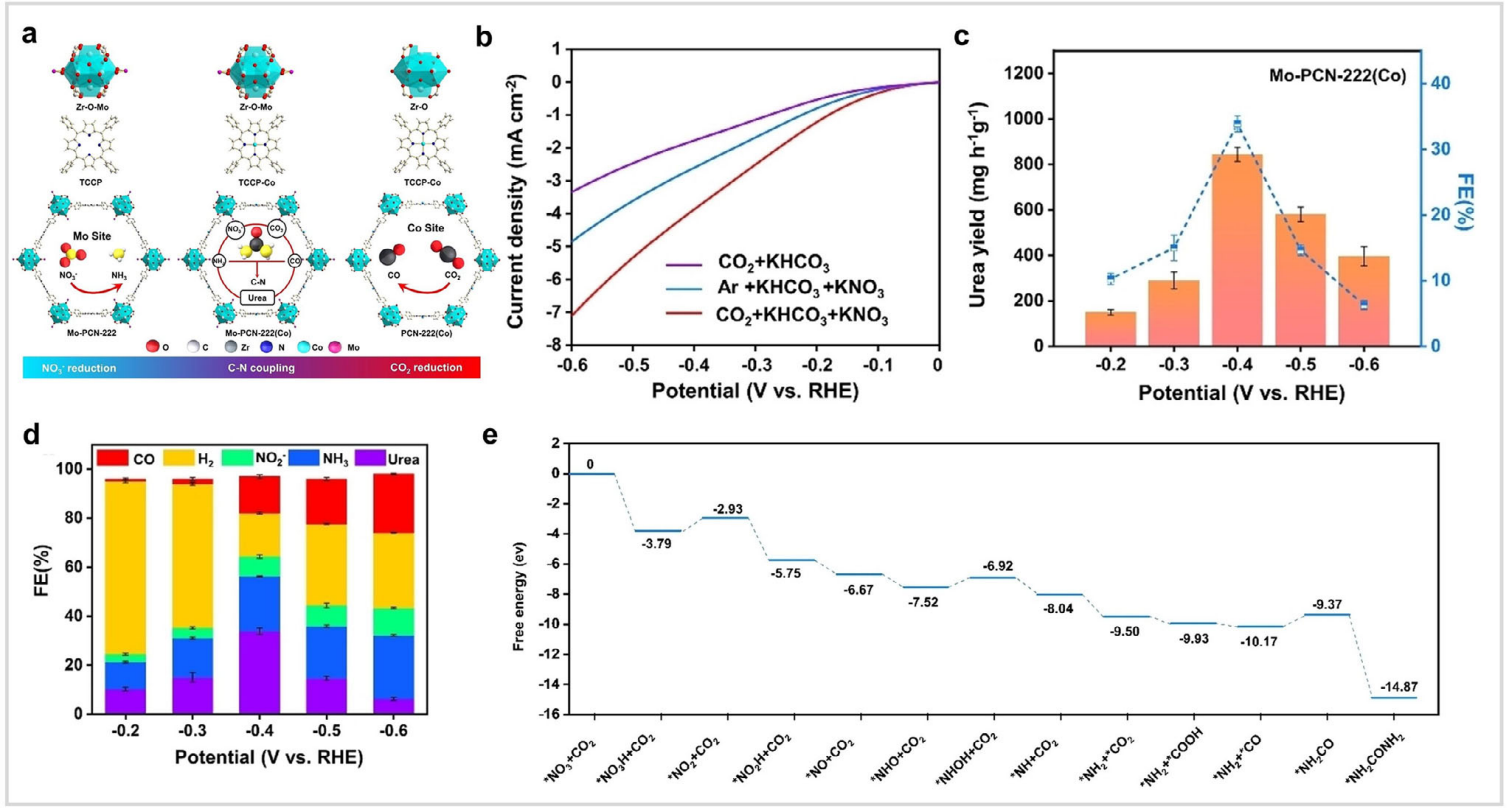
(a) Structural representation of Mo‐PCN‐222/PCN‐222(Co)/Mo‐PCN‐222(Co) crystalline coordination compounds. (b) LSV of Mo‐PCN‐222(Co) under different conditions. (c) Urea yield rates and FE of Mo‐PCN‐222(Co) at various potentials. (d) Product distribution for Mo‐PCN‐222(Co) at different potentials. (e) DFT calculations of the free‐energy diagram. Reproduced with permission.^[^ ^124^ ^]^ Copyright 2024, Wiley‐VCH GmbH.

Although the electrocatalytic synthesis of urea has only attracted the attention of researchers in the past five years, many studies have shown that Por/Pc‐based materials have significant potential. He's group synthesized three MTPPs, namely FeTPP, NiTPP, and CuTPP.^[^ ^123^ ^]^ They tested the catalytic activity of MTPP for C─N coupling by NO_3_^−^ and CO_2_ in a neutral electrolyte. FeTPP demonstrated superior catalytic performance, achieving a maximum urea FE of 19.7%. The electrocatalytic activity of FeTPP was significantly improved by pulse electrolysis, and the urea FE reached 27.7%. They explained that pulse electrolysis can significantly increase the local CO_2_/NO_3_^−^ concentration and reduce the pH, which is conducive to enriching the *CO and *NH_2_ intermediates for C─N coupling. They also synthesized the bimetallic PdCu catalyst and tested its ability to synthesize urea using pulse technology. The urea FE reached 70.36%. Recent investigations have confirmed the potential of MPor‐catalysts in facilitating C─N coupling for the synthesis of urea from NO_3_^−^ and CO_2_. This provides a good reference for subsequent work. Tandem reactions have been shown to be an effective strategy in CO_2_RR but have received little attention in C─N couplings. Based on the above considerations, Wang et al. synthesized a catalyst (Mo‐PCN‐222(Co)) with both CoPor and Mo sites (**Figure** **19**a–e).^[^ ^124^ ^]^ Mo‐PCN‐222(Co) has a clear structure, which is very helpful for studying the catalytic mechanism. They also synthesized Mo‐PCN‐222 and PCN‐222(Co) for comparison purposes. For NO_3_RR, Mo‐PCN‐222 exhibited the best catalytic rate, and the maximum NH_3_ FE reached 80.54%. For CO_2_RR, the maximum CO FE of PCN‐222(Co) reaches about 81.73%. For the C─N coupling from NO_3_^−^ and CO_2_, Mo‐PCN‐222(Co) has better catalytic efficiency compared to Mo‐PCN‐222 and PCN‐222(Co), the maximum urea yield and FE reached 844.11 mg h^−1^ g^−1^ and 33.9%, respectively. The experimental results show that it is effective to construct a tandem catalyst for electrocatalytic synthesis of urea. In addition, they further studied the reaction mechanism of Mo‐PCN‐222(Co) by DFT. The calculation results show that NO_3_^−^ and CO_2_ tend to be adsorbed on Mo and Co surface sites and are reduced to *CO and *NH_2_ intermediates, respectively. The *CO generated at the Co site migrates to the Mo site and couples with *NH_2_ to produce urea. Tandem catalysts have the capability to stabilize the crucial intermediate *CONH_2_ and decrease the energy barrier associated with C─N coupling, thereby enhancing the yield of urea. This study serves as a valuable reference for catalyst design. Compared to catalysts with single sites, this dual‐site catalyst exhibited better catalytic activity in the electrocatalytic synthesis of urea.^[^ ^125^ ^]^

### 5.3 Phthalocyanine‐Based Catalysts For Electrocatalytic Synthesis of Urea

**Figure 20:**
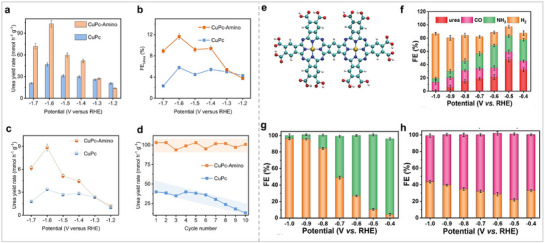
(a) Urea yield rate, (b) FE, and (c) partial current densities of CuPc and CuPc‐Amino. (d) Electrochemical stability tests at −1.6 V versus RHE. Reproduced with permission.^[^ ^127^ ^]^ Copyright 2024, Springer Nature. (e) Schematic model of biCoPc. Products FE of biCoPc catalyst at different potentials in (f) CO_2_‐saturated 0.2 M KHCO_3_ + 0.02 M KNO_2_ solution, (g) Ar‐saturated 0.2 M K_2_SO_4_ + 0.02 M KNO_2_ solution, and (h) CO_2_‐saturated 0.2M KHCO_3_ solution. Reproduced with permission.^[^ ^128^ ^]^ Copyright 2024, Wiley‐VCH GmbH.

MPor has shown good catalytic performance for C─N coupling. MPc and MPor have similar structures, so MPc may also show good performance. Recently, Pickup et al. tested the catalytic activity of MPcs for urea synthesis from NO_2_^−^ and CO_2_.^[^ ^126^ ^]^ The maximum urea FE of CoPc/C was only 3%. When CoPc/C was mixed with ionic liquids, the urea FE reached 27%, and the urea yield was approximately 0.04 mol h^−1^. After modification with an ionic liquid, the catalytic activity of MPc was significantly improved. This may be because the addition of ionic liquid promotes C─N coupling. They also studied the role of the support in the catalytic reaction. The authors replaced the carbon material with Cu‐loaded carbon material to form a catalyst (CoPc/Cu/C) with dual CoPc and Cu sites. The test results showed that the urea FE was approximately 16.1%, and the urea yield was significantly improved (0.095 mol h^−1^). The above work demonstrates the importance of vector selection. Wang et al. modified CuPc with amino groups to synthesize CuPc‐Amino and studied the effects of substituents on their catalytic C─N coupling (**Figure** **20**a–d).^[^ ^127^ ^]^ In the process of synthesizing urea using NO_3_^−^ and CO_2_, the maximum urea yield rate and FE of CuPc were 39.9±1.9 mmol h^−1^ g^−1^ and 5.8%, respectively. Compared with CuPc, the maximum urea yield rate and FE of CuPc‐Amino were significantly improved, reaching 103.1±5.3 mmol h^−1^ g^−1^ and 11.9%, respectively. The urea yield rate of CuPc‐Amino did not decrease significantly after 10 cycles. This also verifies the stability of CuPc‐Amino. However, the urea yield rate of CuPc decreased by 67.4% after 10 cycles. The experimental results show that the presence of amino groups can effectively prevent Cu from leaching from Pc, thereby enhancing the catalytic effect. MPc with a single active site can effectively change the catalytic activity of MPc by regulating the ligand and carrier types. However, the urea FE of MPc is still low. To change this situation, Huo et al. constructed biCoPc with dual active sites through rational design.^[^ ^128^ ^]^ They synthesized urea by co‐reduction using CO_2_ and NO_2_^−^ as C and N sources, respectively. Controlled potential electrolysis experiments show that the urea FE of biCoPc reaches 47% at −0.5V versus RHE in Figure 20f, which is significantly better than that of MPc molecules with a single active site. For NO_2_RR, NH_3_ FE reached 90%. For CO_2_RR, the main products of biCoPc were CO and H_2_, and the CO FE reached 78% at −0.5 V versus RHE. This experimental result shows that biCoPc has catalytic activity for both NO_2_RR and CO_2_RR. This is a prerequisite for electrocatalytic urea synthesis. This also indicates that the construction of Pc catalysts with dual Co sites is effective. The authors explained that the unique planar macromolecular structure of biCoPc and the increase in Co valence promoted the adsorption of carbon and nitrogen sources, thereby promoting C─N coupling to generate urea.

**Figure 21:**
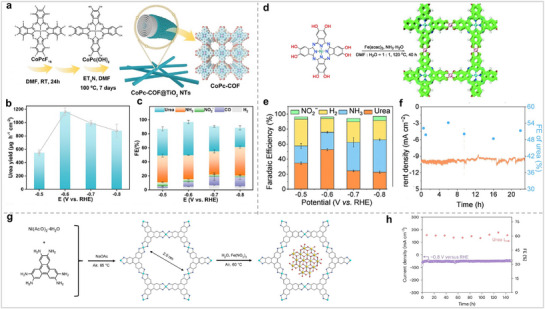
(a) Schematic diagram of CoPc‐COF@TiO_2_ NTs. (b) Urea yield rate and (c) products FE of CoPc‐COF@TiO_2_ NTs. Reproduced with permission.^[^ ^129^ ^]^ Copyright 2023, Springer Nature. (d) The synthesis and structure illustration of PcNi‐Fe‐O. (e) Various products FE of PcNi‐Fe‐O under different potentials. (f) Durability test of PcNi‐Fe‐O at −0.6 V versus RHE. Reprinted from ref. Reproduced with permission.^[^ ^130^ ^]^ Copyright 2024, Wiley‐VCH GmbH. (g) Synthesis route of γ‐Fe_2_O_3_@Ni‐HITP. (h) I‐t curve of γ‐Fe_2_O_3_@Ni‐HITP at −0.8 V versus RHE. Reprinted from ref. Reproduced with permission.^[^ ^131^ ^]^ Copyright 2024, Springer Nature.

Due to the presence of π–π bonds, Pc molecules aggregate during the catalytic process, thereby affecting the catalytic activity. The use of Pc molecules as ligands to construct a framework with a periodic structure can effectively reduce the above problems. Jiang et al. synthesized a CoPc‐COF via a nucleophilic substitution reaction using CoPcF_16_ and CoPc(OH)_8_ as ligands.^[^ ^129^ ^]^ CoPc‐COF@TiO_2_ NTs were produced by facilitating the in situ growth of CoPc‐COF onto a TiO_2_ substrate.(**Figure** **21**a). The authors tested the catalyst's catalytic activity for C─N coupling via NO_3_^−^ and CO_2_. The electrochemical results showed that the maximum urea yield and FE were 1205 µg h^−1^ cm^−2^ and 49%, respectively. They explained that the synergistic effect of CoPc‐COF and TiO_2_ in CoPc‐COF@TiO_2_ promoted the C─N coupling. In situ FT‐IR spectroscopy showed that there were two obvious peaks at 1101 and 1171 cm^−1^, which corresponded to C‐O and H‐N‐H bonds, respectively. This indicates that NO_3_RR and CO_2_RR proceeded simultaneously. Simultaneously, the peak at 1450 cm^−1^ was attributed to C‐N, which is the key evidence for the production of urea. In situ FT‐IR shows that *CO and *NH_2_ are key intermediates for C─N coupling. This also proves once again that constructing a catalyst with a tandem catalytic effect is a very effective strategy. Although the Pc‐catalysts showed good catalytic activity for C─N coupling from NO_3_^−^ and CO_2_, the current intensity of urea was very low, which also resulted in a poor urea yield. Therefore, it is necessary to improve the urea yield while improving the urea selectivity. Recently, Liao et al. synthesized a new MOF (PcNi‐Fe‐O) with dual active sites using NiPc as ligand and square‐planar FeO_4_ as node (Figure 21d–f).^[^ ^130^ ^]^ In the synthesis of urea from NO_3_^−^ and CO_2_, PcNi‐Fe‐O efficiently produced urea (FEurea = 54.1%) at 10.1 mA cm^−2^, which exceeds most reported electrocatalysts. After 20 h of testing, the current density and urea FE did not decrease significantly, indicating that PcNi‐Fe‐O has good stability. To make the electrocatalytic synthesis of urea technology reach the industrial level, they expanded the working electrode area to 25 cm^2^. 20.164 g of urea was produced after running for 8 h, which shows that electrocatalytic synthesis of urea has development potential. Building upon this research, the catalyst was redesigned to enhance the efficiency of the electrocatalytic synthesis of urea. Ultrasmall γ‐Fe_2_O_3_ nanoparticles (< 2 nm) were encapsulated in a metal organic framework (Ni‐HITP) with good conductivity (40 S cm^−1^) to obtain the composite material γ‐Fe_2_O_3_@Ni‐HITP (Figure 21g,h).^[^ ^131^ ^]^ The maximum urea FE was 67.2%, the urea yield was 20.4 (2) g h^−1^ g^−1^_cat_, and the corresponding current density reached 90 mA cm^−2^. Their research has dramatically increased the yield of electrocatalytic synthesis of urea, which establishes a good research basis for future industrial applications. Although Por/Pc‐based catalysts exhibit good catalytic activity for the electrocatalytic synthesis of urea, the urea yield rate is very low. Enhancing the yield of urea through the improvement of the conductivity of Por/Pc‐based catalysts represents a highly valuable research endeavor. The reaction pathway for electrocatalytic urea synthesis was clearly identified, which is the key to catalyst design.

**Figure 22:**
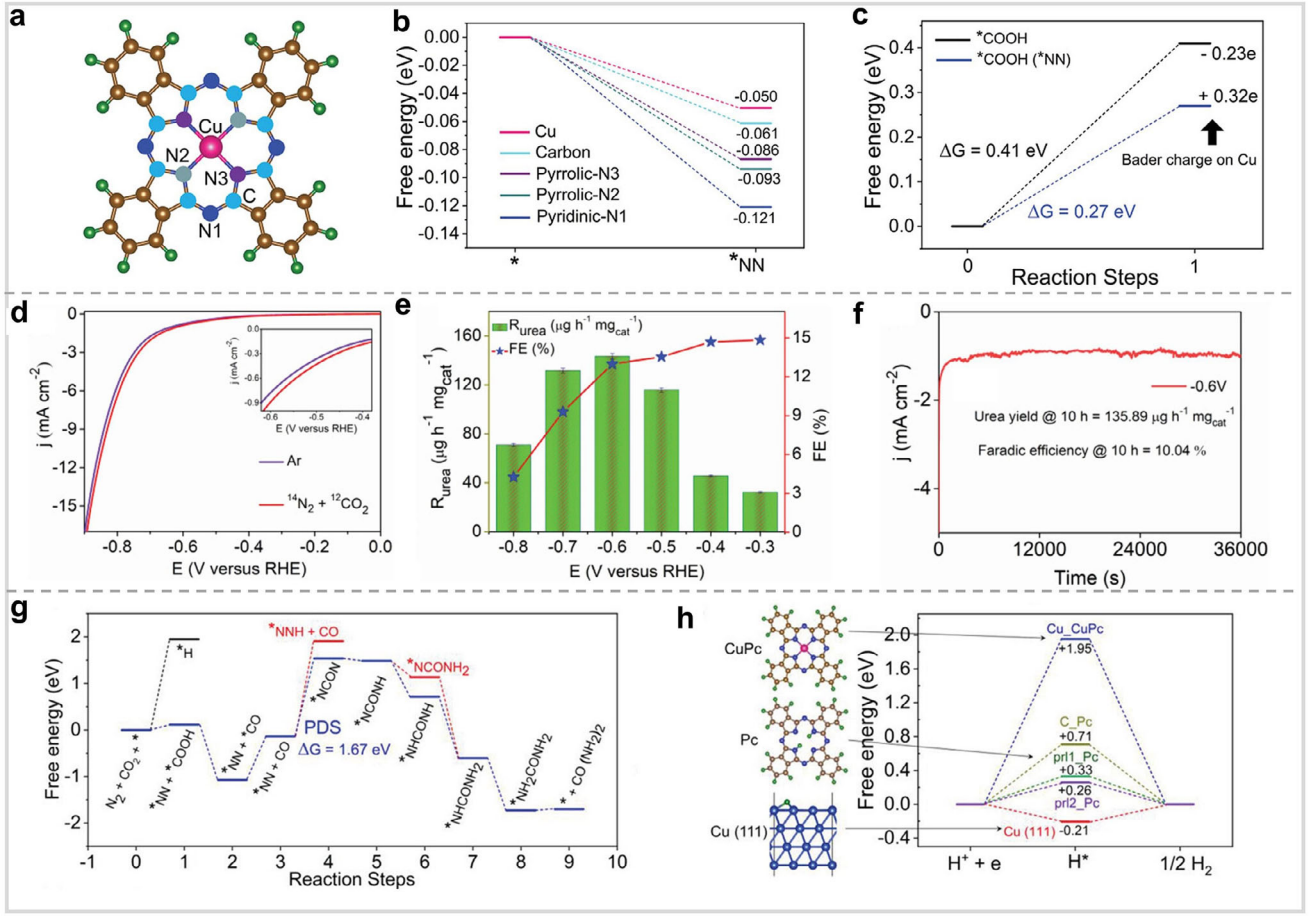
(a) The model of CuPc. (b) Diagrams of free energy for N_2_ adsorption on Cu and pyrrolic‐N in CuPc. (c) Free energy diagram of *COOH adsorption on Cu center of CuPc. (d) LSVs of CuPc NTs 0.1M KHCO_3_. (e) Urea yield rate and FE of CuPc NTs. (f) I–t curves of CuPc NTs. (g) The free energy profile of intermediates for NRR adsorption on Cu site. (h) The free energy diagram of HER on possible active sites of catalysts. Reproduced with permission.^[^ ^132^ ^]^ Copyright 2022, Wiley‐VCH GmbH.

Por/Pc‐catalysts have exhibited remarkable catalytic performance in facilitating the electrocatalytic carbon‐nitrogen coupling reaction between NO_3_^−^ and carbon dioxide CO_2_ for the production of urea. While NO_x_^−^ has been extensively studied as a nitrogen source, research on employing N_2_ for urea synthesis remains limited. Ghorai's group studied the catalytic performance of CuPc for synthesizing urea by N_2_ as N source (**Figure** **22**).^[^ ^132^ ^]^ They considered both the metal center and the pyrrolic‐N as catalytic sites and investigated them through theoretical calculations (Figure 22a,b). The calculation results show that pyrrole‐N1 is more likely to adsorb and activate N_2._ The electrochemical test results (Figure 22e) showed that the FE and yield of urea were 12.99% and 143.47 µg h^−1^ mg^−1^_cat_ at −0.6 V versus RHE, respectively. The experimental results show that pyrrole‐N1 is mainly responsible for the reduction of N_2_, while the Cu site is responsible for the reduction of CO_2_. C–N bonds were detected using in situ Fourier transform infrared spectroscopy (FT‐IR), which is strong evidence for the formation of urea. The reaction mechanism was further studied using theoretical calculations. The calculation results showed that the CO formed on the Cu site was released and then formed *NCON with N_2_. The triple bond of N_2_ was weakened by CO, and the distance between N≡N bonds increases from 1.12 to 1.37 Å. *N_2_ and CO form *NCON, which is the RDS of the entire reaction, and the corresponding ∆G is 1.67 eV. HER is a competing reaction for urea synthesis, so the authors also calculated the hydrogen adsorption on possible active sites. CuPc is more favorable for urea synthesis due to its high ΔG_H_ value (+1.95 eV) for hydrogen adsorption. In this study, the electrocatalytic urea synthesis of CuPc was investigated by combining experiments, in situ characterization, and theoretical calculations. This is very beneficial for the study of reaction mechanisms.

In recent years, the electrocatalytic synthesis of urea using nitrogen‐containing substances and CO_2_ as precursors has attracted considerable attention. Among all nitrogen‐containing substances, NO_2_^−^, NO_3_^−^, and N_2_ are commonly used as nitrogen sources. N_2_ is chosen as a nitrogen source because it is abundant and in line with the strategy of sustainable development. Theoretical and experimental results show that the FE and yield of urea synthesized by electrocatalysis using N_2_ and CO_2_ as raw materials are low. This faces the following challenges. 1) Insufficient CO_2_/N_2_ chemical adsorption on the catalyst surface; 2) the high stability and low solubility of N_2_ (≈941 kJ/mol) lead to a large overpotential, which makes the hydrogen evolution reaction the dominant reaction. Addressing these challenges is crucial for improving urea yield and FE. Compared with the synthesis of urea using N_2_ as the nitrogen source, the yield and FE of urea synthesized by electrocatalysis using NO_2_^−^/NO_3_^−^ as the nitrogen source were greatly improved. This is mainly attributed to the weaker bond energy of N‐O (≈204 kJ/mol) and its high water solubility. At present, the application of MPor/Pc‐based catalysts in electrocatalytic urea synthesis remains to be explored. The catalysts for electrocatalytic urea synthesis are mainly metals and metal oxides. Liu et al. summarized the relevant work on electrocatalytic urea synthesis in recent years and introduced the types of catalysts.^[^ ^133^ ^]^ In the study of electrocatalytic urea synthesis using N_2_ and CO_2_ as raw materials, the FE of urea was mainly concentrated at 5–30%, and the yield was usually less than 1000 µg h^−1^ mg^−1^. When NO_2_^−^/NO_3_^−^ is used as the raw material, the FE of urea is mostly maintained at 30–70% and reaches a maximum of 90%. In addition, the urea yield rate is usually greater than 1000 µg h^−1^ mg^−1^. By comparison, it can be seen that the FE and yield of urea synthesis using NO_3_^−^/NO_2_^−^ as the nitrogen source are significantly higher than those of urea synthesis using N_2_ as the nitrogen source. Electrocatalytic synthesis of urea is still a long way from industrialization. Therefore, catalysts with good catalytic activity and low cost still need to be further developed (**Table** **3**).

**Table 3:** Comparison of the NO_x_^−^/CO_2_ co‐reduction reaction performance of porphyrin/phthalocyanine‐based materials.

Catalysts	Yield [µg h^−1^ mg^−1^]	FE [%]	Electrolyte	Refs.
FeTPP/CNTs	394.9	27.70	0.2 M KHCO_3_ 0.1 M KNO_3_	[123]
ZnO	581.2	54.83	0.2 M KHCO_3_ 0.1 M KNO_3_	[123]
PdCu	1461.1	70.36	0.2 M KHCO_3_ 0.1 M KNO_3_	[123]
Mo‐PCN‐222(Co)	844.1	33.90	0.1 M KHCO_3_ 0.05 M KNO_3_	[124]
CoPc/C/CFP	3.0	27	0.1 M NaHCO_3_ 5×10^−3^M NaNO_2_	[126]
CuPc	2398.0	5.8	0.1 M KHCO_3_ 0.05 M KNO_3_	[127]
CuPc‐Amino	6196.3	11.9	0.1 M KHCO_3_ 0.05 M KNO_3_	[127]
biCoPc	24.6	47.7	0.2 KHCO_3_ 0.02 M KNO_2_	[128]
CoPc‐COF@TiO_2_ NTs	754.3	49	0.3 M KHCO_3_ 0.2 M KNO_3_	[129]
PcNi−Fe−O	2103.5	54.1	0.1 M KNO_3_	[130]
γ‐Fe_2_O_3_@Ni‐HITP	20 434	67.20	1 M KHCO_3_ 0.1 M KNO_3_	[131]
CuPc NTs	143.6	12.99	0.1 M KHCO_3_ N_2_	[132]

## 6 Conclusion and Perspectives

**Figure 23:**
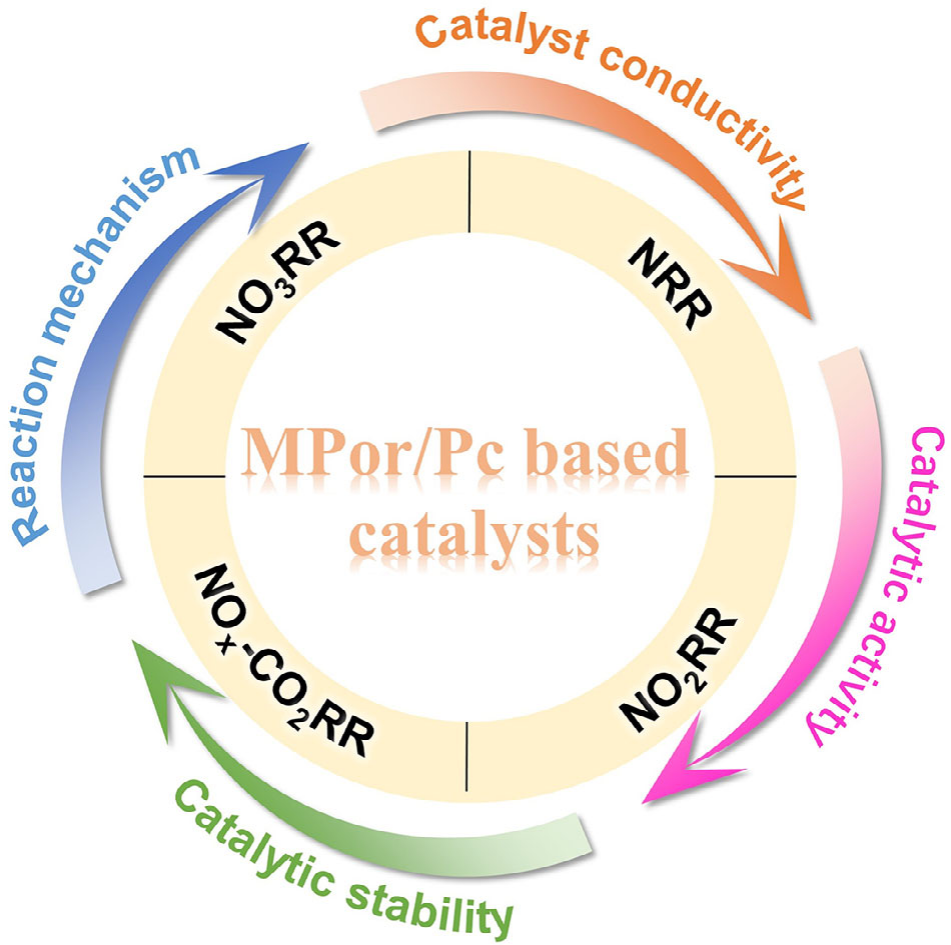
Applications and challenges of Por/Pc‐based catalysts in N conversion‐related reactions.

At present, due to excessive human intervention in nature, the Earth's nitrogen cycle has been affected to a certain extent. Excessive use of nitrogen fertilizers not only damages the ecological environment but also poses a threat to human health. Therefore, using green electricity‐driven electrochemical technology to replace high‐energy‐consuming industries and treat excess nitrogen‐containing pollutants is a potential approach. Since electrocatalytic technology is limited by catalysts, it is still some distance away from commercial application. Por/Pc‐catalysts with adjustable structure, clear active sites, and stable physicochemical properties are ideal catalyst models. Investigating the correlation between the structure of catalysts and their catalytic activity can shed light on the reaction mechanism, thereby offering essential insights for the future design of catalysts. Our research group mainly studies the construction of Por/Pc‐catalysts and their electrocatalysis applications, and has accumulated a certain amount of experience. Therefore, we summarize the modification methods of Por/Pc molecular catalysts, the construction of Por/Pc‐based derivatives, and the application and reaction mechanism of Por/Pc‐based catalysts in electrocatalytic nitrogen fixation (**Figure** **23**). By summarizing the recent applications of MPor/MPc‐based materials in electrocatalytic nitrogen fixation, it was found that Por/Pc‐based catalysts only showed moderate catalytic activity due to their poor conductivity and other disadvantages. MPor/MPc‐based materials still face significant challenges in terms of conductivity, catalytic activity, reaction mechanisms, and stability. To enhance the catalytic performance of Por/Pc‐based catalysts, it is essential to address the aforementioned four aspects.

**(1) Catalyst conductivity**. The catalyst's conductivity plays a pivotal role in influencing its catalytic performance. To increase the product yield, it is necessary to increase the conductivity of the catalyst. Por/Pc‐based catalysts are organic semiconductor materials. The disadvantage of poor conductivity limits its development in the field of electrocatalysis. Currently, interface engineering strategies are the most common and effective means to improve the conductivity of MPor/Pc catalysts. The specific methods of the interface engineering strategies are as follows:

**Carbon material support strategies**. The conductivity of Por/Pc‐based catalysts is a key factor affecting their catalytic activities. One method to improve their conductivity is to combine them with carbon materials (carbon nanotubes, carbon black, graphene, etc.). The most common method is to load Por/Pc‐based catalysts onto carbon nanotubes through π‐π interactions. This method enhances electron transfer while simultaneously mitigating catalyst aggregation. In addition, N, S‐doped carbon materials are also often used as catalyst supports. Because N, S‐doped carbon (such as N‐CNTs) coordinates with the metal center through lone pairs of electrons. This can effectively regulate the d‐band center of metals and accelerate interfacial electron transfer. Similar to N, S‐doped carbon materials, carbon materials with vacancies and defects as carriers can also improve the conductivity of Por/Pc‐based catalysts. Although the above method can improve the conductivity of Por/Pc‐based catalysts, the catalysts adsorbed on carbon materials by π‐π interactions are not robust. The Por/Pc‐based catalyst still aggregates during the reaction. Some researchers have used materials with special functional groups as carriers to connect Por/Pc‐based catalysts via chemical bonds. This method can significantly increase conductivity while also effectively preventing catalyst aggregation. This method is highly dependent on the number of functional groups on the carriers. The number of MPor/Pc catalysts covalently linked to the support is typically small. Although the above methods have different disadvantages, they are still effective in improving the conductivity of catalysts.

In addition to carbon materials, metals and metal oxides are often used as carriers. For example, Burdyny et al. modified Fe‐porphyrin on a Ni support to construct a heterogeneous catalyst.^[^ ^134^ ^]^ They believe that the interaction between the molecular catalyst and metal can stabilize the oxidation state of the molecular catalyst, and enable continuous electron transfer. DFT calculations revealed that the distance between Fe and Ni carrier was less than 2.00 Å. There is an electron density transfer from Fe to Ni, which can reduce the orbital energy. In conclusion, the conductivity and dispersion of MPor/Pc‐based catalysts can be effectively increased using highly conductive supports.

**Building a conductive network**. Designing the structure of MPor/Pc catalysts can also improve their conductivity.^[^ ^135^ ^]^ For example, Zhou et al. described a conductive metallophthalocyanine‐based NiPc‐CoTAA framework with cobalt(II) tetraaza[14]annulene linkages.^[^ ^136^ ^]^ This highly conjugated structure and overlapping phthalocyanine units provide NiPc‐CoTAA with advantages such as high electrical conductivity (0.52 S m^−1^) and carrier mobility (0.15 cm^2^ V s^−1^). Currently, there are abundant ligands used to synthesize highly conductive organic frameworks. Ligands with high π conjugation (such as phenylene, perylenetetracarboxylic diimide, etc.) can reduce the resistance to carrier migration by delocalizing π electrons. Ligands with planar rigidity can reduce electron scattering caused by intramolecular vibrations and enhance intermolecular π‐π stacking. In addition, ligands with redox groups, heteroatom doping, and high polarizability can be used in combination with MPor/Pc to improve conductivity. The above method was used to construct a framework with high conductivity through a molecular engineering strategy without changing the MPor/Pc structure. However, some researchers have found that calcination can effectively enhance the conductivity of MPor/Pc catalysts. This is because calcination can transform the MPor/Pc catalyst into metal nitrogen‐carbon materials (MNCs). The high conductivity of MNCs is essentially the result of the synergy of the graphitized carbon matrix, the metal‐nitrogen coordination center, and the heteroatom doping effect. This design not only optimizes the electron transfer path but also achieves a balance between conductivity and catalytic function through atomic‐level regulation.

These methods can effectively improve the conductivity of Por/Pc‐based catalysts, providing strong support for their application in electrocatalytic nitrogen fixation.

**(2) Catalytic activity**. Although Por/Pc‐based catalysts have shown good catalytic performance in corresponding reactions, there is still a certain gap compared with excellent catalysts, such as precious metal catalysts, alloy catalysts, etc. Due to the interaction between Por/Pc molecules, the Por/Pc molecular catalysts will aggregate during the catalytic process, thereby affecting their catalytic activity. Improving the catalytic activity of MPor/Pc‐based catalysts can be regulated by the following aspects.

**Center metal**. Different metals have different characteristics, and their adsorption on intermediates is obviously different. Therefore, the choice of central metal is particularly important. For example, the Por/Pc‐based catalysts with Cu sites showed better catalytic activity and better adsorption of NO_3_^−^ than other catalysts for the NO_3_RR. This is because the d‐orbital electrons of Cu can effectively interact with the π* antibonding orbital of NO_3_^−^, promoting the adsorption and activation of NO_3_^−^. In addition, Cu metal is also beneficial to promote C‐C coupling to produce multi‐carbon products in the carbon dioxide reduction reaction (CO_2_RR). Feng et al. studied the catalytic activity of MPc‐pz with different metals (M = Ni, Co, Zn, Mn, Fe, and Cu) for NRR.^[^ ^76^ ^]^ Among them, FePc‐pz exhibits significantly higher catalytic activity than other MPc‐pz. This also illustrates the importance of metal center selection for MPor/Pc. Since electrocatalytic synthesis of urea involves CO_2_RR and nitrogen reduction reactions, constructing a Por/Pc‐based catalyst with dual metal sites is a better choice.

**Substituent modification**. Substituent modification strategies have been shown to be effective in regulating the catalytic performance of MPor/Pc molecules.^[^ ^137^ ^]^ The modification of substituents can regulate the charge distribution of MPor/Pc molecules. This can effectively affect the adsorption of MPor/Pc to intermediates, and ultimately achieve the purpose of regulating catalytic activity. For example, electron‐donating/electron‐withdrawing substituents can significantly change the charge distribution of MPor/Pc molecules, thereby affecting the adsorption capacity of the active sites for intermediates. Besides, there are also acidic/basic groups and charged functional groups with positive/negative charges, etc. In addition to changing the charge distribution of MPor/Pc, they have unique effects. In NO_3_RR, positively charged substituents can also increase the adsorption of NO_3_^−^ on MPor/Pc and promote the dissociation of NH_4_^+^ through Coulombic forces. The strong interaction between MPor/Pc leads to its aggregation and reduces the exposure of active sites, which ultimately affects the catalytic performance. Therefore, the introduction of large steric groups can inhibit the aggregation of MPor/Pc molecules. Furthermore, axial ligands such as pyridine and thiol are introduced to modulate the electronic state of the metal. This modulation subsequently influences the adsorption energy of the metal on the intermediate, ultimately enhancing catalytic activity. In addition to MPor/Pc molecular catalysts, other MPor/Pc‐based derivative materials can introduce functional groups to regulate catalytic activity. MPor/Pc‐based MOFs can introduce functional groups, such as ionic liquids and hydroxyl groups, at sites such as organic ligands or metal clusters. MPor/Pc‐based organic frameworks can introduce functional groups into ligands. In conclusion, it is very effective to adjust the catalytic activity of the catalyst by introducing substituents according to the characteristics of the catalyst and reaction.

**Dispersion of catalytic sites**. The dispersion of active sites is an important factor affecting the catalytic activity of the catalyst. Highly dispersed active sites can effectively increase the electrochemical active area and contact more reaction substrates. The intermolecular forces between the MPor/Pc molecules cause them to aggregate and affect the dispersion of the active sites. As mentioned above, MPor/Pc molecules can be adsorbed on the surface of CNTs through π–π interactions. This not only increases their conductivity but also effectively increases the exposure of the active sites. The covalent attachment of MPor/Pc molecules to the surface of CNTs is also an effective means of increasing their dispersion. In addition, some researchers have confined MPor/Pc molecules to porous materials to increase their dispersibility. The above strategies combine MPor/Pc molecules with other materials to increase their dispersibility. Constructing MPor/Pc‐based derivative materials is also a feasible route for increasing the dispersion of active sites. For example, MPor/Pc can be integrated into the organic framework as a building block. In conclusion, the above strategy was proven to be very effective for the dispersion of MPor/Pc‐based catalysts.

The above three strategies are very effective strategies to optimize the catalytic activity of MPor/Pc. In addition, constructing composite materials and optimizing test conditions can also effectively regulate the catalytic activity of MPor/Pc. For example, the appropriate electrolyte, reaction temperature, and electrolytic cell type are selected according to the reaction characteristics and the characteristics of the catalyst.

**(3) Catalytic stability**. Stability is an important parameter for evaluating catalysts and is a necessary condition for large‐scale applications. Currently, the stability of Por/Pc‐based catalysts in N‐cycle‐related reactions has only been tested for tens of hours or over multiple cycles. However, this is still a long way from commercial application. To improve catalyst stability, it is important to prevent not only aggregation of the catalyst, but also destruction of the catalyst or deactivation of the active sites. This requires a catalyst that is structurally stable and resistant to corrosion. It is a very effective method to construct Por/Pc into an organic framework through covalent bonds. This is because, compared with other connection methods, connections through covalent bonds are more stable. To avoid the corrosion of the catalyst and removal of metal sites, a suitable electrolyte can be selected, such as a neutral electrolyte. The central metal of MPor/Pc also has the risk of dissolution during the reaction. For example, the Cu in CuPor will dissolve during the reaction and reach a dynamic equilibrium, and the dissolved Cu may even form Cu clusters. Selecting axial ligands containing strong coordinating atoms such as N, S, and O (such as pyridine and thiol) can effectively prevent metal dissolution. Protecting MPor/Pc through a carrier is also an important means to improve stability. Encapsulation of MPor/Pc in MOFs (such as ZIF‐8) or mesoporous silica channels. In conclusion, there are many strategies to improve the stability of MPor/Pc. It is very important to choose the right strategy while maintaining the catalytic activity of MPor/Pc.

**(4) Reaction mechanism**. The study of reaction mechanisms is important for understanding catalytic reactions. This is very helpful for researchers to understand the reactions and design catalysts. For example, it reveals the step‐by‐step pathways of reactions, including intermediates and transition states. It explains how and why reactions occur, helping to predict outcomes under different conditions. This helps minimize unwanted byproducts and improve the yield and purity. Enables the design of better catalysts (homogeneous, heterogeneous, or enzymatic) by identifying the rate‐limiting steps. At present, operando detection technology and theoretical calculations have become indispensable for studying reaction mechanisms. Online differential electrochemical mass spectrometry (DEMS), operando Fourier transform infrared (operando FT‐IR), operando Raman, and operando X‐ray absorption fine structure (operando XAFS) are commonly used to study the reaction mechanisms. DEMS is a highly sensitive technology for the real‐time monitoring of gas‐phase products or volatile intermediates in electrochemical reactions. Its core lies in combining an electrochemical system with a mass spectrometer and eliminating background interference through differential design. Operando FT‐IR spectroscopy is a technique that monitors the chemical structure and molecular dynamics of samples in real time during reactions or other changes. It can track the evolution of intermediates, products, and functional groups in chemical reactions and reveal the reaction mechanisms. Operando Raman is similar to operando FT‐IR. It is also a nondestructive analysis technology that monitors the molecular vibration, lattice structure, and chemical bond information of samples in real time during reactions or environmental changes. By detecting changes in the intermediates and functional group information using the above in situ techniques, the relevant reaction pathways can be inferred. For example, Sheng et al. studied the reaction pathway of TiO_2_−C using CO_2_ and NO_3_^−^ for C─N coupling to synthesize urea by online DEMS, operando FT‐IR, and Raman spectroscopy.^[^ ^138^ ^]^ Operando XAFS can detect the local atomic structure and electronic state evolution of materials in real time under reaction conditions. Its core lies in analyzing the coordination environment, valence state, and dynamic change process of the target element through the fine structure of the X‐ray absorption edge. The above operando characterization not only provides the changes of intermediates during the reaction but also enables a good understanding of the changes in the catalyst. The corresponding reaction path can be inferred based on the test results and verified through theoretical calculations. The combination of theoretical calculation and operando characterization has become an important means to study the reaction mechanism.

In conclusion, we reviewed the construction of Por/Pc‐based catalysts and their applications in electrocatalytic nitrogen fixation. Porous carbon‐based catalysts have shown good potential. In catalytic reactions, the selectivity of specific products was close to 100% when Por/Pc‐based materials were used as catalysts. Simultaneously, the application of Por/Pc‐based catalysts is very helpful for studying reaction mechanisms. However, there are still many challenges. We hope that this study contributes to the application of Por/Pc‐based catalysts in electrocatalytic nitrogen fixation.

## Conflict of Interest

The authors declare no conflict of interest.

## Data Availability

UK Research and Innovation (UKRI includes AHRC, BBSRC, ESRC, EPSRC, Innovate UK, MRC, NERC, Research England, STFC).
